# Induction of Monoterpenoid Oxindole Alkaloids Production and Related Biosynthetic Gene Expression in Response to Signaling Molecules in *Hamelia patens* Plant Cultures

**DOI:** 10.3390/plants13070966

**Published:** 2024-03-27

**Authors:** Ana Luisa López-Vázquez, Edgar Baldemar Sepúlveda-García, Elizabeth Rubio-Rodríguez, Teresa Ponce-Noyola, Gabriela Trejo-Tapia, Josefina Barrera-Cortés, Carlos M. Cerda-García-Rojas, Ana C. Ramos-Valdivia

**Affiliations:** 1 Departamento de Biotecnología y Bioingeniería, Centro de Investigación y de Estudios Avanzados del Instituto Politécnico Nacional (CINVESTAV-IPN), Ciudad de Mexico 07360, Mexico; analuisa.lopez@cinvestav.mx (A.L.L.-V.); tponce@cinvestav.mx (T.P.-N.); jbarrera@cinvestav.mx (J.B.-C.); 2 Laboratorio de Biotecnología Vegetal, Instituto de Biotecnología, División de Estudios de Posgrado, Universidad del Papaloapan, San Juan de Tuxtepec 68301, Oaxaca, Mexico; esepulveda@unpa.edu.mx; 3 Departamento de Biotecnología, Centro de Desarrollo de Productos Bióticos, Instituto Politécnico Nacional (CEPROBI-IPN), Yautepec 62730, Morelos, Mexico; lizrubio6@gmail.com (E.R.-R.); gttapia@ipn.mx (G.T.-T.); 4 Departamento de Química, Centro de Investigación y de Estudios Avanzados del Instituto Politécnico Nacional (CINVESTAV-IPN), Ciudad de Mexico 07360, Mexico; ccerda@cinvestav.mx

**Keywords:** *Hamelia patens*, monoterpene indole alkaloids, oxindole alkaloids, salicylic acid, jasmonic acid, tetrahydroalstonine synthase, strictosidine β-glucosidase

## Abstract

*Hamelia patens* (Rubiaceae), known as firebush, is a source of bioactive monoterpenoid oxindole alkaloids (MOAs) derived from monoterpenoid indole alkaloids (MIAs). With the aim of understanding the regulation of the biosynthesis of these specialized metabolites, micropropagated plants were elicited with jasmonic acid (JA) and salicylic acid (SA). The MOA production and MIA biosynthetic-related gene expression were evaluated over time. The production of MOAs was increased compared to the control up to 2-fold (41.3 mg g DW^−1^) at 72 h in JA-elicited plants and 2.5-fold (42.4 mg g DW^−1^) at 120 h in plants elicited with SA. The increment concurs with the increase in the expression levels of the genes *HpaLAMT*, *HpaTDC*, *HpaSTR*, *HpaNPF2.9*, *HpaTHAS1*, and *HpaTHAS2.* Interestingly, it was found that *HpaSGD* was downregulated in both treatments after 24 h but in the SA treatment at 120 h only was upregulated to 8-fold compared to the control. In this work, we present the results of MOA production in *H. patens* and discuss how JA and SA might be regulating the central biosynthetic steps that involve *HpaSGD* and *HpaTHAS* genes.

## 1. Introduction

*Hamelia patens* Jacq. (Rubiaceae), known as firebush, hummingbird bush and chacloco, is a perennial shrub native to the southeastern region of Mexico and Central America, widely known for its uses in traditional medicine. Several pharmacological properties have been attributed to their phytochemical extracts, such as antimicrobial [1], immunostimulant [2], anti-inflammatory [3], cytotoxic [4,5], and antihyperglycemic activities [6]. 

In *H. patens*, specialized metabolites such as triterpenes, flavonoids, and alkaloids have been identified. Some of these alkaloids possess pharmacological importance, which has generated a wide number of patents, and some of them are also present in the *Uncaria* genus [7]. *H. patens* plants produce the monoterpene indole alkaloids (MIAs) tetrahydroalstonine, aricine (10-methoxytetrahydroalstonine), and aricine *N*-oxide, together with the monoterpenoid oxindole alkaloids (MOAs) pteropodine, isopteropodine, speciophylline, 10-hydroxypteropodine, 10-hydroxyisopteropodine (rumberine), 10-methoxypteropodine (hameline), and 10-methoxyisopteropodine (palmirine) [8,9].

The pentacyclic MOA biosynthesis in *H. patens* is still unclear. However, it has been previously suggested that, in the species *Mitragyna parvifolia*, tetrahydroalstonine is the MIA precursor of the MOA pteropodine [10]. This was confirmed in *H. patens* leaves [9] by feeding the plants with radioactive tryptophan, which was biosynthetically transformed into MIAs and their corresponding MOA.

The biosynthesis of MIAs has been extensively studied in the species *Catharanthus roseus* (Apocynaceae), in which it has been demonstrated that the pathway operates through a multi-step enzyme network that is highly regulated by environmental and developmental factors [11]. The MIA biosynthesis starts from two main biosynthetic pathways. The first one is the shikimic acid pathway, where tryptophan is converted by the enzyme tryptophan decarboxylase (TDC) to tryptamine, the indole precursor of MIAs. In the second one, the methyl-erythritol phosphate (MEP) pathway yields loganic acid, which is transformed into loganin by the enzyme loganic acid methyltransferase (LAMT) [12]. Loganin is then converted by secologanin synthase (SLS) to secologanin [13], the terpene precursor of MIAs. The enzyme strictosidine synthase (STR) condenses tryptamine and secologanin to strictosidine, the central common precursor to all MIAs. The strictosidine is exported from the vacuole to the cytosol by a nitrate/peptide (NPF) family transporter located in the *C. roseus* tonoplast (CrNPF2.9), and the following steps on MIA biosynthesis depend on this transference [14]. Then, the enzyme strictosidine β-glucosidase (SGD) located in the nucleus [15] transforms strictosidine into a highly reactive ring-opened strictosidine aglycone that converts spontaneously to a variety of isomers [16], such as cathenamine [17]. 

The MIA tetrahydroalstonine is formed from cathenamine by the enzyme tetrahydroalstonine synthase (THAS) of the medium chain dehydrogenase/reductase (MDR) protein family [16]. In *C. roseus* and *Mitragyna speciosa*, these enzymes are co-localized in the nucleus and interact with SGD [18,19]. In *M. speciosa*, a single protein localization assay in yeast revealed that MspSGD was localized in the nucleus and MspTHAS candidates were localized in the cytoplasm. However, when MspSGD was co-expressed with MspTHAS, it was re-localized to the nucleus entirely in the presence of MspSGD [19].

Regarding the MIA conversion to an MOA, a recent study on *M. speciosa* reported a cytochrome P450 enzyme from the CYP71 family (MsCYP72056) responsible for the formation of the tetracyclic spirooxindole alkaloids 3-*epi*-corynoxeine and isocorynoxeine from the indole alkaloid hirsuteine [20]. 

It has been reported that MOAs represent an essential defense for the plant against environmental factors. They are produced as a response to oxidative stress, associated with biotic and abiotic factors that lead to an increase in their production [21]. Derived from this knowledge, the use of elicitors as a tool to create a differential stimulus for biosynthetic pathways has allowed for studying their effect on metabolic regulation mechanisms [22]. The elicitors are external substances or factors that induce physiological responses in the plants that lead to the regulation of genes involved in the biosynthetic pathways of specialized metabolites. These genes are induced by signaling molecules that unchain a complex transcriptional regulatory network [23]. The signaling molecules salicylic acid (SA), jasmonic acid (JA), and its jasmonate derivatives have been used to study the regulation of alkaloid biosynthesis in plants. In *U. tomentosa* (Rubiaceae) root cultures, MOA production and the transcription of *STR* and *SGD* genes were enhanced by a combination of JA and buthionine sulfoximine [24]. *M. speciosa* shoot cultures treated with MeJA 10 µM for 24 h elicited mitragynine accumulation, while *TDC* and *STR* expression levels were enhanced [25]. JA induces several TFs in *C. roseus*, such as the ORCA3, 4, and 5, which regulate MIA pathway genes *TDC*, *STR,* and *SGD* [26,27], and the TFs BIS1 and BIS2, which regulate iridoid pathway genes [28].

SA plays an important role during the plant defense response against biotic and abiotic stress, such as pathogen infections, salinity, and drought, by transcribing different sets of defense genes controlled by SA-mediated mechanisms [29]. Furthermore, SA participates in gene-mediated defense and systemic acquired resistance (RSA) in plants [30] and interacts with other signaling pathways, such as JA [31]. In *U. tomentosa* plants, 1 µM SA promoted an increase in MOA production [32]. The exogenous application of SA stimulated MIAs in *C. roseus* [33] and regulated the transcription of early genes of the *C. roseus* MIA biosynthetic pathway, such as *TDC*, *SLS*, and *STR*, after 24 h of application [34,35]. 

**Figure 1:**
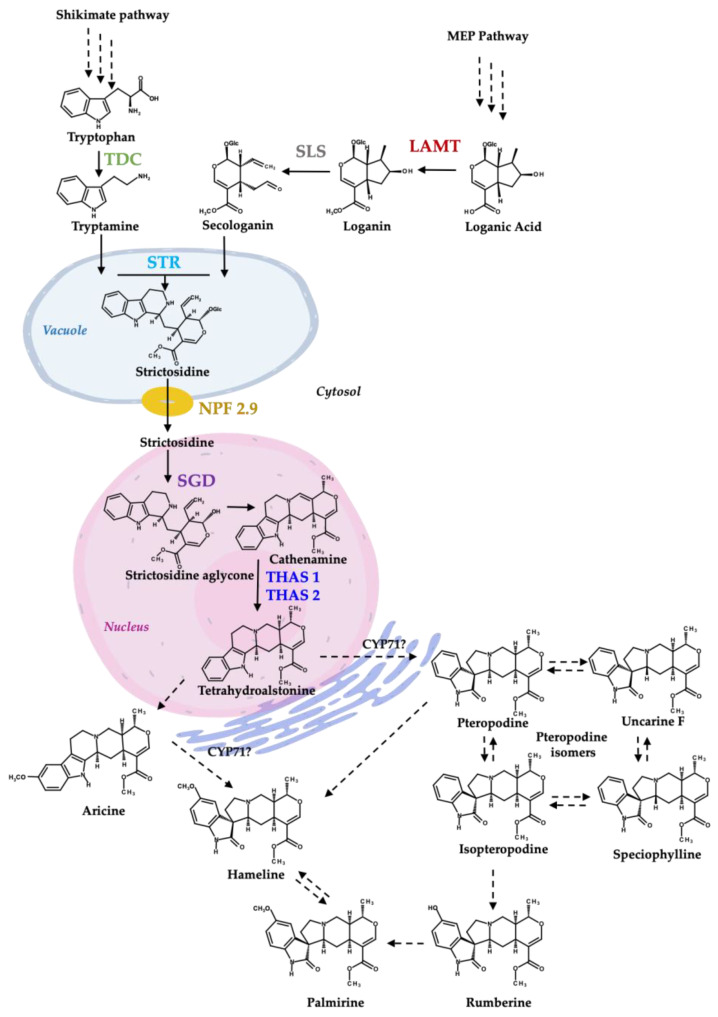
Proposed biosynthesis of MIAs and MOAs in *H. patens* (Modified from [9]). TDC—tryptophan decarboxylase (green), LAMT—loganic acid methyltransferase (red), SLS—secologanin synthase (grey—not evaluated in this work), STR—strictosidine synthase (light blue), NPF2.9—nitrate/peptide family strictosidine transporter (yellow), SGD—strictosidine β-glucosidase (purple), THAS1 and THAS2—tetrahydroalstonine synthase 1 and 2 (blue). CYP71-P450—CYP71 family spirooxindole synthases involved in MIA conversion to an MOA [20]. The biosynthetic steps from shikimate pathway and iridoids pathway to tetrahydroalstonine are based on *C. roseus* MIA biosynthesis.

Most medicinal plants are not model plant species, and the biosynthesis and regulation of these metabolites still need to be fully understood. Few studies of the MOA biosynthetic pathway can be found in *H. patens* [9]. In this study, we report the cloning of *H. patens* genes involved in the biosynthesis of MIAs, the precursors of MOAs (Figure 1), and their expression levels in SA- and JA-elicited plants associated with MOA production. Based on our results, a gene regulation profile under each treatment is proposed.

## 2. Results and Discussion

### 2.1. Phylogenetic Tree Analysis of the Cloned Partial Sequences 

**Figure 2:**
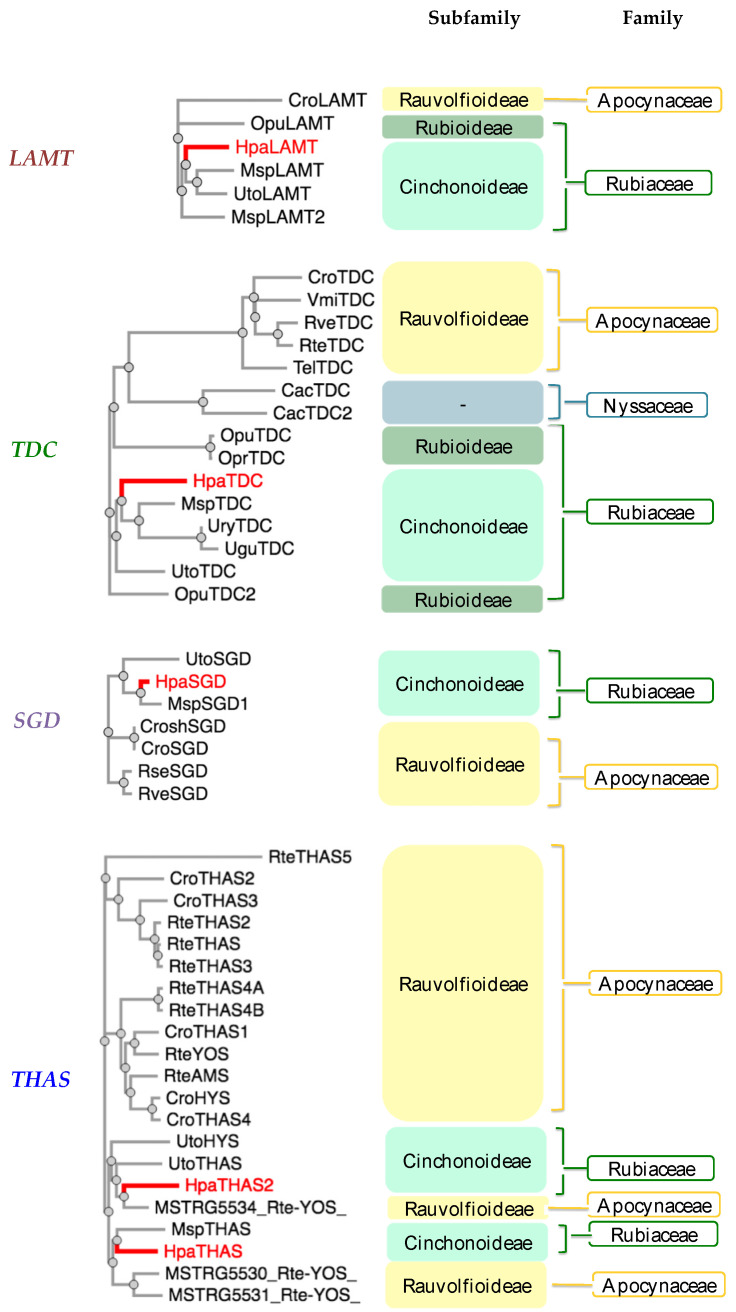
Phylogenetic analysis of monoterpenoid indole alkaloid (MIA) biosynthetic genes from *Hamelia patens* cloned in this study (labeled in red). Plant species included: *Cac*—*Camptotheca acuminata*, *Cro*—*Catharanthus roseus*, *Msp*—*Mitragyna speciosa*, *Opr*—*Ophiorrhiza prostata*, *Opu*—*Ophiorrhiza pumilla*, *Rse*—*Rauvolfia serpentina*, *Rte*—*Rauvolfia tetraphylla*, *Rve*—*Rauvolfia verticillata*, *Tel*—*Tabernaemontana elegans*, *Ugu*—*Uncaria guianensis*, *Ury*—*Uncaria rhynchophylla*, *Uto*—*Uncaria tomentosa*, *Vmi*—*Vinca minor*. Plant families: Rubiaceae (Green), Apocynaceae (Yellow), and Nyssaceae (Blue). MIA pathway genes: *TDC* (tryptophan decarboxylase), *LAMT* (loganic acid methyltransferase), *SGD* (strictosidine β-glucosidase), *THAS* (tetrahydroalstonine synthase), *YOS* (yohimbane synthase). The amino acid sequences are included in Supplementary Material S3.

**Figure 3:**
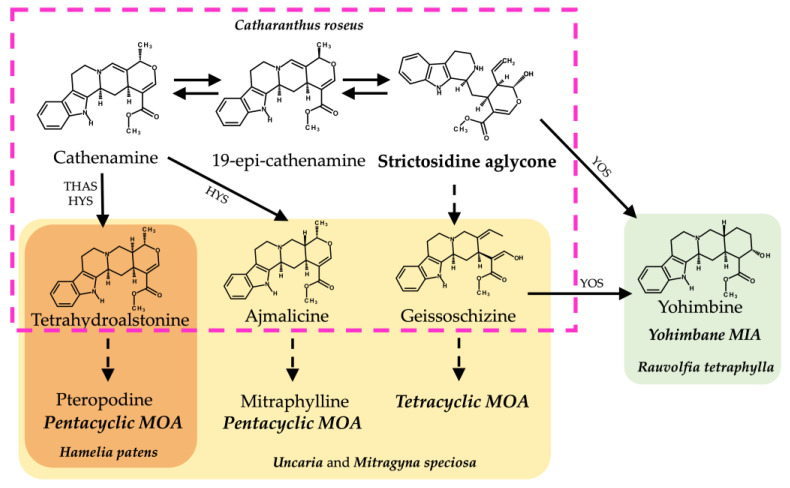
Biosynthetic pathways from strictosidine aglycone of related MIAs and MOAs in different alkaloid-producing plant species. Structures inside the discontinuous pink lines belong to the *C. roseus* MIA biosynthetic pathway. Structures in yellow shading belong to the proposed MOA biosynthesis pathways in *Uncaria* and *M. speciosa.* Structures in orange shading belong to the proposed MOA biosynthesis in *H. patens.* Structures in green fill belong to the yohimbane biosynthesis in *R. tetraphylla.* Abbreviations: THAS (tetrahydroalstonine synthase), HYS (heteroyohimbine synthase), YOS (yohimbane synthase).

The amino acid sequences from the cloned MIA-related genes from *Hamelia patens HpaLAMT*, *HpaTDC*, *HpaSGD*, *HpaTHAS1*, and *HpaTHAS2* were analyzed against other gene sequences from MIA-producing species (listed in Supplementary Material S3). The *HpaLAMT*, *HpaTDC*, and *HpaSGD* genes were classified closer to other Rubiaceae species genes, as several conserved regions were found mainly with sequences of *Mitragyna speciosa* and *Uncaria* species (Figure 2), which are also MOA-producing species that belong to the subfamily Cinchonoideae [36]. Inside the Rubiaceae cluster, the early biosynthetic genes *HpaLAMT* and *HpaTDC* are separated from *M. speciosa* and *Uncaria*. The differences among the gene sequences of these species with respect to *H. patens* genes suggest a possible divergence of *M. speciosa* and *Uncaria* from a common ancestor with *H. patens*, which might have led to the alkaloid variability in the species. While *M. speciosa* and *Uncaria* produce tetracyclic MOAs derived from geissoschizine and pentacyclic MOAs derived from the heteroyohimbines tetrahydroalstonine and ajmalicine [37,38], *H. patens* only produces pentacyclic MOAs derived from tetrahydroalstonine (Figure 3) [9]. 

The *HpaTHAS1* gene was shown to be more identical to *MspTHAS1* and *HpaTHAS2* to the MSTRG5534 gene from *Rauvolfia tetraphylla* (Apocynaceae), capable of synthesizing multiple yohimbanes from the alkaloid geissoschizine [39] (Figure 3). Both *HpaTHAS1* and *HpaTHAS2* were clustered with the Rubiaceae *THAS* genes [16,18,19] and with *R. tetraphylla* MDR genes MSTRG5530, MSTRG5531, and MSTRG5534 [39]. This is very interesting since *H. patens* has not been reported to produce yohimbine-type alkaloids or geissoschizine-derived alkaloids [8]. A similar classification occurred with *R. tetraphylla*, where the yohimbane synthase RteYOS that catalyzes the biosynthesis of the stereoisomers yohimbane, rauwolscine, and corynanthine was found to be more identical to *C. roseus CroTHAS1* than to any other *R. tetraphylla*, with YOS activity reported in that work [39].

### 2.2. Alkaloid Production after SA and JA Elicitation 

The effect of the elicitors JA and SA in MOA production was analyzed. The concentration of JA was selected based on the previous work of [9], where 500 µM JA showed an increase in MIA and MOA total content as well as an upregulation of some MIA genes. The SA concentration was chosen from the results of a previous experiment where SA at 0, 10, 50, and 100 µM were tested, and the highest MOA production was achieved at 50 µM after 120 h (Figure S2). 

**Figure 4:**
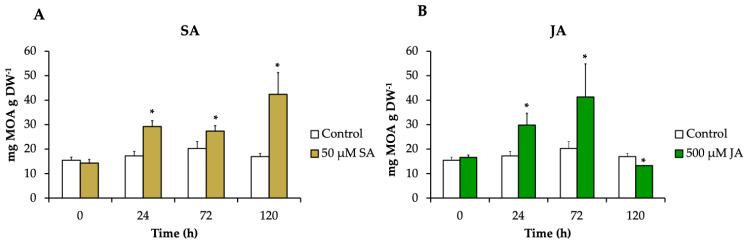
The MOA content in *H. patens* leaves after elicitation with (**A**) 50 μM of SA (yellow bars), (**B**) 500 μM of JA (green bars), and control plants treated with 30% EtOH (white bars). Error bars indicate standard error from the mean (*n* = 3). (*) Significant differences to the control based on *t*-test (*p* ≤ 0.05).

The alkaloids pteropodine, isopteropodine, speciophylline, uncarine F, rumberine, and hameline were identified and quantified in each treatment. *H. patens* leaves elicited with SA and JA significantly increased the alkaloid production compared to the control up to 2.5-fold (42.4 mg g DW^−1^) and 2-fold (41.3 mg g DW^−1^), respectively. The MOA increment started at 24 h in both treatments, and the highest alkaloid production in SA-elicited plants occurred at 120 h and in JA-elicited plants at 72 h (Figure 4). 

Our results showed that SA and JA had a significant effect on the accumulation of total MOAs in *H. patens* leaves. Both elicitors promoted similar alkaloid production levels.

### 2.3. MIA Pathway Gene Expression

**Figure 5:**
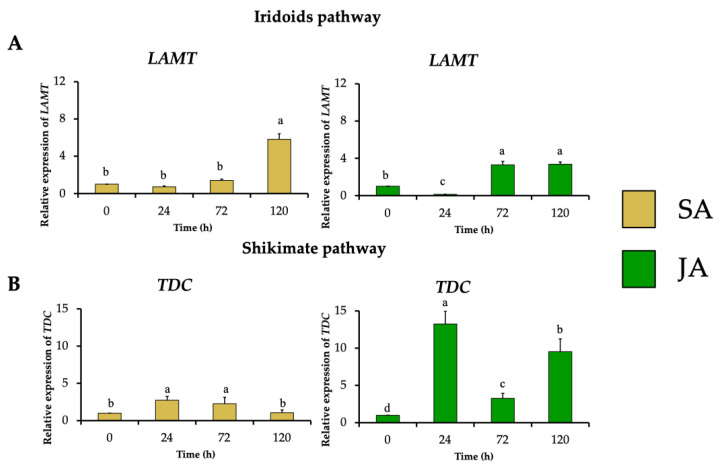
Effect of SA (yellow bars) and JA (green bars) on the relative expression of genes involved in early steps of in MIA biosynthesis in *H. patens*, over time. (**A**) *LAMT* (loganic acid methyltransferase) from the iridoids pathway and (**B**) *TDC* (tryptophan decarboxylase) from the shikimate pathway. Error bars indicate standard error from the mean (*n* = 3). Different letters indicate significant differences (*p* ≤ 0.05) based on Tukey’s test. The assignment of the letters (a–d) corresponds to the alphabetical order from the highest to lowest relative expression values for each graphic.

The effect of the elicitors JA and SA on the expression profile of MIA biosynthetic genes was investigated in *H. patens*. From the terpene biosynthetic pathway, *HpaLAMT* expression levels increased to 5.8-fold under SA elicitation and 3.3-fold under JA elicitation at 120 and 72 h, respectively, coinciding with the time of highest MOA production (Figure 5A). The shikimate pathway gene *HpaTDC* had the highest relative expression at 24 h in SA and JA treatments with 2.7-fold and 13.2-fold, respectively (Figure 5B). The upregulation of *HpaTDC* occurred before the highest accumulation of MOAs in each treatment. This coheres with this gene’s participation in the early steps of MIA biosynthesis to produce tryptamine, and together with secologanin, are condensed by STR to form strictosidine, the central precursor to MIA biosynthesis [40]. In *C. roseus* hairy roots, JA has proven to promote the accumulation of MIAs by inducing TFs such as MYC2, which regulate the early genes of the shikimate pathway, such as *TDC* [27]. In *C. roseus* cell suspension cultures, JA induced BIS1 and BIS2, which regulate iridoid pathway upstream *LAMT* genes [28]. SA has also regulated the transcription of the early genes of the MIA biosynthesis pathway of *C. roseus* leaves, such as *TDC*, *SLS*, and *STR*, after 24 h of application [34,35].

**Figure 6:**
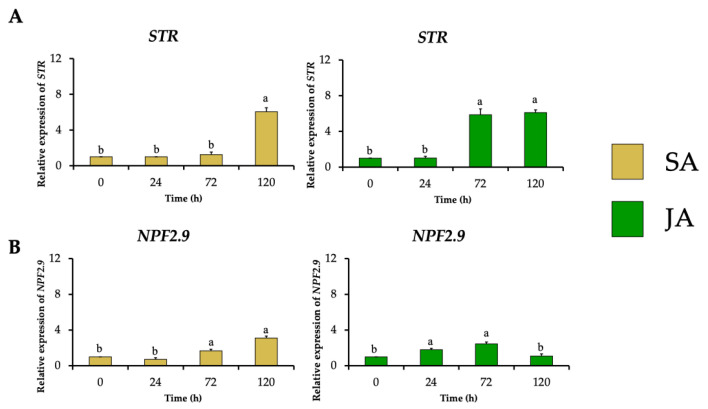
Effect of SA (yellow bars) and JA (green bars) on the relative expression of genes involved in the biosynthesis and transport of the common MIA precursor strictosidine in *H. patens* over time. (**A**) *STR* (strictosidine synthase) and (**B**) *NPF2.9* (nitrate/peptide strictosidine transporter). Error bars indicate standard error from the mean (*n* = 3). Different letters indicate significant differences (*p* ≤ 0.05) based on Tukey’s test. The assignment of the letters (a,b) corresponds to the alphabetical order from the highest to lowest relative expression values for each graphic.

The relative expression of the gene encoding strictosidine synthase (*HpaSTR*) increased to 6-fold and 5.9-fold after SA and JA treatments with the highest MOA production, respectively (Figure 6A). This increment was followed by stimulating the expression of the strictosidine transporter gene (*HpaNPF2.9*) to 3.1-fold and 2.5-fold, respectively (Figure 6B), which could be necessary for the migration of strictosidine from the vacuole towards the cytosol where the MIA biosynthetic pathway continues as occurs in *C. roseus* [14].

**Figure 7:**
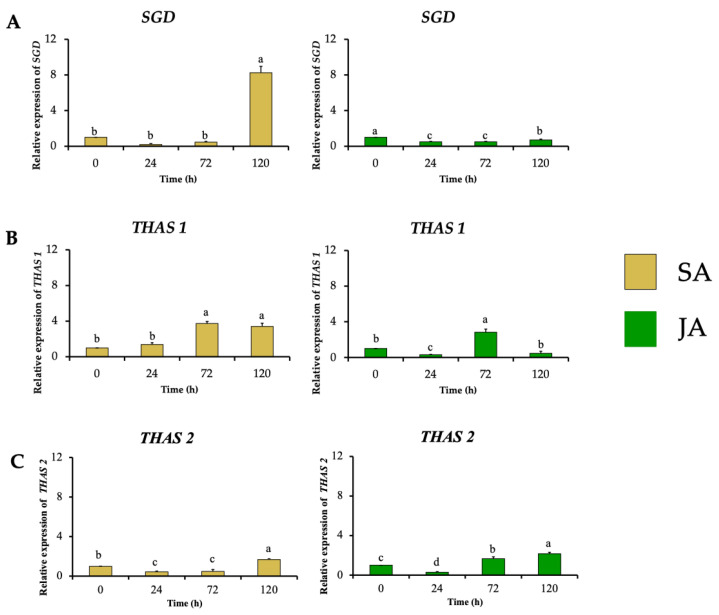
Effect of SA (yellow bars) and JA (green bars) on the relative expression of nuclear genes involved in MIA biosynthesis in *H. patens* over time. (**A**) *SGD* (strictosidine β-glucosidase), (**B**) *THAS1* (tetrahydroalstonine synthase 1), and (**C**) *THAS2* (tetrahydroalstonine synthase 2). Error bars indicate standard error from the mean (*n* = 3). Different letters indicate significant differences (*p* ≤ 0.05) based on Tukey’s test. The assignment of the letters (a–d) corresponds to the alphabetical order from the highest to lowest relative expression values for each graphic.

In the next biosynthetic central step of the conversion of strictosidine to strictosidine aglycone by SGD, *HpaSGD* decreased its expression by 0.7-fold after JA treatment, and no increment in the relative expression compared to the control was found over time (Figure 7A), contrasting with the increment on alkaloid production. A similar effect happened during SA treatment on *HpaSGD*, where no upregulation after elicitation was observed; nevertheless, at 120 h, an increment of 8.2-fold in the gene relative expression of *HpaSGD* occurred (Figure 7A). In a previous study, *H. patens* plants elicited with JA 1 mM increased the production of MOAs. However, such treatment did not promote an increase in the enzymatic activity or transcription of *HpaSGD*, which the authors proposed as one of the control points in the biosynthesis of MOAs in *H. patens* [9], though no biosynthetic downstream genes were evaluated to support this hypothesis. 

This study evaluated the transcription of *HpaTHAS*, which is involved in the next biosynthetic step after HpaSGD. The *THAS*-like genes *HpaTHAS1* and *HpaTHAS2* showed upregulated expression in both elicitor treatments; *HpaTHAS1* under SA treatment presented the highest expression levels of 3.7-fold at 72 h, which was maintained until 120 h. When JA was used, *HpaTHAS1* increased its relative expression to 2.8-fold at 72 h (Figure 7B). The *HpaTHAS2* gene was upregulated 1.7-fold and 2.2-fold in SA and JA treatments, respectively (Figure 7C). These results showed that *HpaTHAS1* had higher levels of expression compared to *HpaTHAS2*. Furthermore, the phylogenetic analyses showed that *HpaTHAS1* was closer to the *M. speciosa MspTHAS* gene, while *HpaTHAS2* resembled an *R. tetraphylla* gene involved in yohimbanes biosynthesis (Figure 2). Therefore, these results indicate that *HpaTHAS1* is the most probable candidate gene that participates in the biosynthesis of tetrahydroalstonine, the MIA precursor of MOAs in *H. patens*. Nevertheless, *HpaTHAS2* could also be involved in this bioconversion step, as has been reported in *C. roseus* and *R. tetraphylla*, where several THAS enzymes catalyze the tetrahydroalstonine biosynthesis [18,39].

**Figure 8:**
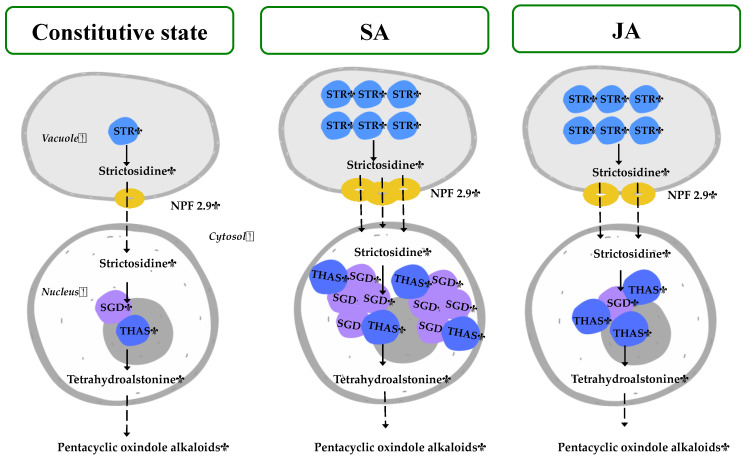
Proposed regulation of *H. patens* MIA biosynthetic genes under SA and JA elicitation for MOA production. The number of colored genes corresponds to the relative expression under each treatment. STR—strictosidine synthase, NPF2.9—nitrate/peptide strictosidine transporter, SGD—strictosidine β-glucosidase, THAS—tetrahydroalstonine synthase.

Figure 8 represents the gene expression results under JA and SA elicitors, in which the MOA biosynthesis in *H. patens* appears to be dependent on *HpaSTR* expression for the biosynthesis of strictosidine and its transport out of the vacuole by HpaNPF2.9. 

On the central step of strictosidine conversion, the *HpaSGD* gene conserved its constitutive expression levels under JA treatment, but to promote the biosynthesis of MOAs, an increase in *HpaTHAS* expression levels was needed. This may be required to promote the re-localization of the *HpaTHAS* gene towards the nucleus to interact with *HpaSGD*, as occurs at the protein level in *M. speciosa* [19] when expressed in yeast and *C. roseus* cells [16]. In the case of the SA treatment, *HpaTHAS1* expression levels increased at 72 h before the highest MOA production occurred and thus subsequently might have boosted *HpaSGD* transcription at 120 h. The above result suggests that the activation of *HpaSGD* also depends on *HpaTHAS1* expression and that a nuclear metabolon might be formed to rapidly metabolize the strictosidine aglycone to tetrahydroalstonine [15,18] and then continuing with MOA biosynthesis. On the other hand, in *C. roseus*, the regulation of this central step has been demonstrated to be influenced by a shorter isoform of SGD (CroshSGD) that lacks β-glucosidase activity and co-exists with *CroSGD* at a constant expression profile. However, this interaction disrupts SGD multimers, which inhibit the SGD activity and impact the MIA biosynthesis downstream by interrupting the recruitment of CroTHAS1 in the cell nucleus [41]. A similar regulation of SGD could be occurring in *H. patens* under constitutive conditions, while SA elicitation might be generating an unbalanced ratio of SGD isoforms. This altered condition could lead to the overexpression of *HpaSGD* and, consequently, to the interaction with HpaTHAS, thus continuing with an enhanced MOA biosynthesis.

Both elicitors, JA and SA, had a similar effect on MOA accumulation and the expression of *Hpa-LAMT*, *Hpa-STR*, and *Hpa-NPF2.9* in *H. patens*. However, the elicitors demonstrated to have a different regulation mechanism in the central step of MIA biosynthesis where *Hpa-SGD* and *Hpa-THAS* are involved, which are the proposed immediate upstream steps before MOA biosynthesis in *H. patens* (Figure 1). These results showed the different forms of regulation that can occur under SA and JA signaling. Both molecules can modulate MIA-biosynthetic gene expression in *H. patens*, which is directly related to the accumulation of MOAs. Our study gives an overview of the possible mechanisms of the JA and SA signaling molecules in regulating MOA biosynthesis; however, further studies are needed to characterize the function of the genes and their regulation in *H. patens.* It is important to consider that no downstream genes involved in MOA biosynthesis were evaluated in our research, which can be also regulated by SA and JA through different mechanisms. 

Salicylates and jasmonates can work antagonistically or synergistically to each other and facilitate plants immunity to provide resistance against stress [42]. The SA and JA signaling pathways are activated after the stimulation of the effector-triggered immunity (ETI), a form of innate immunity in plants generated by specific recognition between pathogen effectors and their corresponding plant cytosolic immune receptors [43]. The activation of ETI leads to the transcriptional upregulation of defense-related genes controlled by SA and JA pathways, which are activated in distinct concentric domains simultaneously in the same plant [44]. This could explain the different gene regulation profiles observed in *H. patens*-elicited plants despite having a similar effect at the final MOA production. 

## 3. Materials and Methods

### 3.1. Plant Material

Plant material, ripe fruits, and dry fruits of *H. patens* were collected from a single specimen in Cuernavaca, Morelos, Mexico (2017). Disinfection and germination of *H. patens* seeds were carried out in a modified Nitsch and Nitsch [45] medium (NNm) described by [8]. The seedlings were propagated by nodal segments and placed in PlantCon™ boxes (MP Biomedicals, Solon, OH, USA) with 100 mL of NNm medium, maintained in a 16/8 h photoperiod of light and darkness (white light lamps, 1000–1300 lx) at a temperature of 25 ± 1 °C. These plants (Figure S1) were used for all the experiments described below. 

### 3.2. SA and JA Elicitation Treatments 

Two-month-old *H. patens* micropropagated plants were elicited with 500 μM JA or 500 μL of 50 μM SA (dissolved in 30% EtOH) by spraying on all the leaves and with 30% EtOH as control. The leaves of the elicited and control plants were collected in liquid nitrogen at 0, 24, 72, and 120 h after treatments and stored at −80 °C for subsequent analysis.

### 3.3. Total RNA Extraction 

RNA extraction was performed using TRIzol™ (Life Technologies Corporation, Invitrogen™, Carlsbad, CA, USA) following the instructions of the supplier, and additional RNA cleaning was performed with the RNeasy MinElute Cleanup Kit (Qiagen™, Germantown, MD, USA). Total RNA was quantified with Nanodrop 2000 (Thermo Scientific™, Wilmington, DE, USA), and its integrity was verified by agarose denaturing gel. Total genomic DNA contamination was removed by RNAse-free DNAse I (Thermo Scientific™, Vilnius, Lithuania) treatment according to the instructions of the supplier. RevertAid reverse transcriptase (RT) (Thermo Scientific™, Vilnius, Lithuania) performed reverse transcription for cDNA synthesis. 

### 3.4. Database Mining and Gene Identification

SRA files (Short Read Archive) and reported transcriptome data from MIA- and MOA-producing plant species belonging to the Apocynaceae, Rubiaceae, and Nyssaceae families were downloaded from different sources and are listed in Table S1. The reads in SRA files were assembled with DNASTAR Lasergene 15 (DNASTAR Inc., Madison WA, USA) to generate the corresponding databases. With this assembled data and the transcriptomes, a unique file was generated. 

The amino acid sequences from *C. roseus* genes: *CroLAMT* (ABW38009.1), *CroTDC* (CAA47898.1), *CroSTR* (CAA43936.1), *CroSGD* (AAF28800.1), *CroNPF2.9* (AQM73449.1) *CroTHAS* (AKF02528.1), *CroHYS* (ANQ45225.1) were downloaded from the NCBI GenBank (Table S2) and used as query to perform a tBLASTn using Bioedit 7.0.4 software with a threshold of E > 10^−10^. 

### 3.5. Design of Degenerate Primers

The nucleotide sequences obtained in the tBLASTn with high identity with *CroLAMT*, *CroTDC*, *CroSTR*, *CroSGD*, *CroNPF2.9*, *CroTHAS*, and *CroHYS* were aligned in ClustalW included in Bioedit 7.0.4. software. Manual editing and detection of conserved regions were carried out to design degenerate primers (Table S3) that amplified at least 200 bp, and the melting temperature (Tm) calculation was performed at http://www.biophp.org (Free Software Foundation, Inc., Boston, MASS, USA) accessed on 1 October 2017. 

### 3.6. Touchdown PCR and Cloning of Partial Sequences of the MIAs Genes

PCR 30 μL final volume was performed with 2 μL of the cDNA (from total RNA of seedlings treated with JA for 48 h, as described above): 0.2 mM dNTPs, 2 μM each of the pair of degenerate primers, 3 μL of 5× buffer, 0.3 μL “Phusion High-Fidelity DNA Polymerase” (Thermo Scientific™, Vilnius, Lithuania). Touchdown PCR [46] was performed with the following parameters: initial denaturation temperature 98 °C for 2 min, followed by 25 cycles of 98 °C for 15 s, 50–40 °C with a decrease of 0.4 °C per cycle, 72 °C for 45 s, followed by 17 cycles of 98 °C per 15 s, 40 °C for 15 s, 72 °C for 45 s, and a final extension of min at 72 °C for 2 min.

PCR products were separated by electrophoresis 1% agarose gel with TBE 1X, and the observed bands with the expected molecular weight were isolated by centrifugation [47]. The purified products were cloned into the pJET1.2 vector using the “CloneJET PCR Cloning Kit (Thermo Scientific™, Vilnius, Lithuania) by *Escherichia coli* DH5-α electroporation with the Gene Pulser Xcell^TM^ electroporation system (1 mm, 165, 208; 1.8 kV, 25 μF, 200 Ω) (Bio-Rad Laboratories, Hercules, CA, USA). Colony PCR was performed with pJET1.2 primers, and positive colonies from each gene were grown in LB liquid medium supplemented with ampicillin 100 μg μL^−1^. Plasmid extraction was performed with the “GeneJET Plasmid Miniprep” kit (Thermo Scientific™, Vilnius, Lithuania) and sequenced with pJET1.2 primers in ABIPRISM 3100 Genetic Analyzer (Applied Biosystems, 3130XL, Foster City, CA, USA). Primers for Rapid Amplification of cDNA Ends (RACE) 3′ (Table S4) were designed to obtain the 3′ extremes of *HpaLAMT*, *HpaTDC*, and *HpaTHAS1*, and for *HpaLAMT* RACE 5′ only, it was performed to obtain the entire sequence. The RACE 3′ and 5′ was performed using the GeneRacer™ Kit (Life Technologies Corporation, Invitrogen™, Carlsbad, CA, USA) for cDNA synthesis, PCR, and cloning in the pCR™4-TOPO™ TA vector following the instructions of the supplier. The cloned products of *HpaLAMT* (OL436237.1), *HpaTDC* (OL436239.1), *HpaSGD* (PP157909), *HpaNPF2.9* (OL436238.1), *HpaTHAS1* (OL436240.1), and *HpaTHAS2* (OL436241.1) (Supplementary Material S2) were sequenced as described above. The phylogenetic analysis of the obtained *H. patens* genes sequences *HpaLAMT*, *HpaTDC*, *HpaSGD*, *HpaTHAS1*, and *HpaTHAS2*, together with those previously reported in the literature (Supplementary Material S3), was performed on https://www.ebi.ac.uk/jdispatcher/ accessed on 29 November 2023 [48]. 

### 3.7. Design of Oligonucleotides for qPCR Assays

Primer designs for qPCR (Table S5) were performed from the obtained cloned sequences of *Hpa-LAMT*, *HpaTDC*, *HpaSGD*, *HpaTHAS1*, *HpaTHAS2*, and *HpaNPF2.9*. The *HpaEF1α* sequence was obtained from the transcriptome database of *Hamelia patens* [49]; for *HpaSTR*, an alignment of reported sequences of *Mitragyna speciosa* (EU288197.1), *Ophiorrhiza pumila* (AB060341.1), *Ophiorrhiza japonica* (EU670747.1), and *Uncaria rhynchophylla* (OL310251.1; ON125563.1) was performed, and a highly conserved region was used for the design of primers. The design was performed in http://primer3plus.com accessed on 7 November 2017 [50], applying the default parameters of the website with modifications: Amplicon size: 80 to 200 bp, Size of primer (b): Minimum; 20, Optimum; 25, Maximum; 28, Tm (°C): Minimum; 62, Optimum; 64, Maximum; 68, % GC: Minimum; 35, Optimum; 65, Maximum; 80. For the determination of secondary structures and dimerization, the website of “Beacon Designer Free Edition” (http://www.premierbiosoft.com, San Francisco, CA, USA, accessed on 7 November 2017) was used, and we selected the pair of oligonucleotides with values of ΔG > −6. 

### 3.8. Gene Expression Analysis by RT-qPCR

The PCR reactions with a final volume of 12 μL were performed using 0.8 μg of cDNA (obtained as described above) as a template and specific primers for each gene at a final concentration of 0.16 μM with Maxima SYBR Green/ROX™ qPCR kit (Thermo Scientific™, Vilnius, Lithuania). The RT-qPCR was performed using the CFX Real-time PCR Detection System (Bio-Rad™, Hercules, CA, USA) under the following conditions: 50 °C for 2 min for one cycle, followed by 95 °C for 10 min for one cycle, then 40 cycles with denaturation at 95 °C for 15 s, alignment at 60 °C for 15 s, and extension at 72 °C for 30 s. A fusion curve and electrophoresis run were performed to confirm the formation of a single amplification product. To determine expression levels, three reference genes 60SRP-P0, 40SRP-S6, and elongation factor 1 (EF1-α) stability were verified by RT-PCR and agarose 1% gel electrophoresis. Among them, EF1-α gene was the most stable and was used as the housekeeping gene to normalize the expression values of each studied gene. Each gene was determined out in triplicate and gene relative expression values were determined as described by [51]. The efficiency values (E) and Ct were obtained by submitting the data to LinRegPCR (Version 2021.1, Amsterdam AMC, The Netherlands).

### 3.9. Total MOA Extraction and Quantification 

Alkaloid extraction was carried out according to the protocol used by [9] with some modifications. The leaves of each sample were pulverized in the presence of liquid nitrogen, and 150 mg of tissue was used for extraction. The alkaloids were extracted with 0.5 mL of MeOH-H_2_O 1:1, vortexed for 15 s, and placed in a sonication bath for 10 min. Afterwards, the samples were centrifuged at 5000 rpm for 5 min. The supernatant of each sample was separated in a new tube and filtered through a 0.45 μm nylon membrane. The filtered samples were injected into a piece of HPLC-PAD equipment (Varian Chromatograph ProStar 333, Walnut Creek, CA, USA) with a reverse-phase column of 250 mm × 4.6 mm (Waters^TM^ Spherisorb^®^, Milford, MA, USA), and the mobile phase was composed of acetonitrile (A) and phosphate buffer pH 7 (B). The elution proceeded at a rate of 0.7 mL/min as described next: first, at an isocratic flow with 40% A for 70 min, then a linear gradient at 45% A for 5 min, and finally, isocratic with 45% of A for 65 min, with a total time of 105 min. The detection was carried out at 244 nm, obtaining the UV spectra with a diode array from 190 to 400 nm, a chromatogram of the *H. patens* extract is included in Supplementary Material S5. To identifying the alkaloids, standards previously isolated from the plant were injected [8]. To quantify MOAs, a calibration curve with pteropodine was used [9].

### 3.10. Statistical Analysis

All experiments were conducted in triplicate, and mean values with standard deviation are reported. The results were subjected to a one-way analysis of variance (ANOVA). Significant differences in mean values among treatments were determined with Tukey’s test and Student’s *t*-test with a level of significance of *p* ≤ 0.05.

## 4. Conclusions

Our study showed the effect of the elicitors SA and JA on the upregulation of MIA biosynthesis-related genes, leading to an increase in MOA production. SA and JA have two different approaches to regulate the MIA biosynthetic genes in *H. patens*, mainly in the central step involving SGD and THAS genes. The obtained results can be used to increase the production of MOAs in *H. patens* and to understand the regulation of their biosynthesis when acting as plant chemical defenses. 

## Data Availability

All data generated during this study are included in this published article and in the supplementary material file. The nucleotide sequences of the evaluated genes were deposited in the GenBank database with the following accession numbers: *Hamelia patens* loganic acid O-methyltransferase mRNA, complete cds (*Hpa*LAMT)-OL436237.1, *Hamelia patens* putative tryptophan decarboxylase mRNA, partial cds (*Hpa*TDC)-OL436239.1, *Hamelia patens* putative transporter exports strictosidine mRNA, partial cds (*Hpa*NPF2.9)-OL436238.1, *Hamelia patens* putative strictosidine beta glucosidase mRNA, partial cds (*Hpa*SGD)- PP157909, *Hamelia patens* putative tetrahydroalstonine synthase mRNA, partial cds (*Hpa*THAS1)-OL436240.1, *Hamelia patens* putative heteroyohimbine synthase (in this work named tetrahydroalstonine synthase 2), partial cds (*Hpa*THAS2)-OL436241.1.

## Supplementary Materials

The following supporting information can be downloaded at: https://www.mdpi.com/article/10.3390/plants13070966/s1 [52,53,54,55,56,57,58,59,60].
