# Supplementary material for: Induction of Monoterpenoid Oxindole Alkaloids Production and Related Biosynthetic Gene Expression in Response to Signaling Molecules in Hamelia patens Plant Cultures
Source: Plants (Basel). 2024 Mar 27;13(7):966. doi: 10.3390/plants13070966 (PMC11013434; doi:10.3390/plants13070966)

Supplementary material

Supplementary material 1.

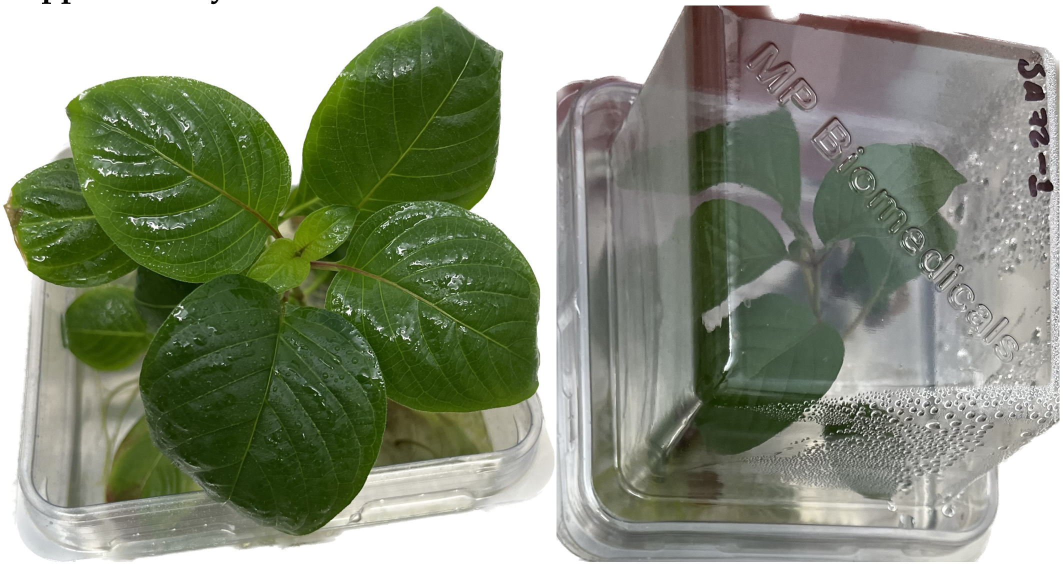

Figure S1. *H. patens* micropropagated plants used for the elicitation experiments.

Table S1. Downloaded files of transcriptomes and gene sequences of MIA-producing plants.

Plant species	Prefix	Family	Accession number	Transcriptome source	Ref
FASTA files					
Amsonia hubrichtii	*Ahu*	Apocynaceae	SPRX096091	PhytoMetaSyn (www.phytometasyn.ca)	[52]
Cinchona ledgeriana	*Cle*	Rubiaceae	-	PhytoMetaSyn (www.phytometasyn.ca)	-
Vinca minor	*Vmi*	Apocynaceae	-	PhytoMetaSyn (www.phytometasyn.ca)	-
Tabernaemontana elegans	*Tee*	Apocynaceae	SPRX096093	PhytoMetaSyn (www.phytometasyn.ca)	[52]
Rauwolfia serpentina	*Rsa*	Apocynaceae	-	http://medicinalplantgenomics.msu.edu/	[53]
Camptotheca acuminata	*Caa*	Nyssaceae	-	http://medicinalplantgenomics.msu.edu/	[53]
Ophiorrhiza pumila	*Opa*	Rubiaceae	-	http://ngs-data-archive.psc.riken.jp/pub/ophiorrhiza_pumila/download/	[54]
Catharanthus roseus	*Cro*	Apocynaceae	-	http://medicinalplantgenomics.msu.edu/	[53]
Short Read Archive (SRA) files					
Rhazya stricta	*Rsi*	Apocynaceae	SRP028239	Genbank NCBI (www.ncbi.nlm.nih.gov)	[55]
Hamelia patens	*Hpa*	Rubiaceae	SRP079620	Genbank NCBI (www.ncbi.nlm.nih.gov)	[49]
Uncaria rhynchophylla	*Ury*	Rubiaceae	SRX362039	Genbank NCBI (www.ncbi.nlm.nih.gov)	[56]

Mitragyna speciosa	*Msp*	Rubiaceae	SRX2855178	Genbank NCBI (www.ncbi.nlm.nih.gov)	[57]

Table S2. MIA-biosynthesis gene sequences

Species	Gene	Code	Accession Nº GenBank	Bibliography
Catharanthus roseus	Loganic Acid Methyl-Transferase	*CroLAMT*	ABW38009.1	[12]
	Tryptophan decarboxylase	*CroTDC*	CAA47898.1	[58]
	Strictosidine synthase	*CroSTR*	CAA43936.1	[59]
	Strictosidine beta-glucosidase	*CroSGD*	AAF28800.1	[17]
	Tetrahydroalstonine synthase	*CroTHAS*	AKF02528.1	[16]
	Heteroyohimbine synthase	*CroHYS*	ANQ45225.1	[18]
	Nitrate/Peptide Family Transporter	*CroNPF2.9*	AQM73449.1	[14]
	Elongation factor 1 -alpha	*CroEF1-α*	ABV58644.1	[49]
Mitragyna speciosa	Strictosidine synthase	*MspSTR*	EU288197.1	-
Ophiorrhiza pumila	Strictosidine synthase	*OpuSTR*	AB060341.1	[54]
Ophiorrhiza japonica	Strictosidine synthase	*OjaSTR*	EU670747.1	[60]
Uncaria rhynchophylla	Strictosidine synthase	*UrySTR*	OL310251.1<br>ON125563.1	-

Table S3. Degenerated primers for *H. patens* genes amplification

Gene	Primer name	Primer Sequence (5´→3´)
LAMT	LAMT-F1deg	THGYDGAYTTNGGNTGYTCNDBHGG
	LAMT-R2deg	ADNRTANDNNHHNAHGTTGAANGARTC
TDC	TDC-F1deg	GARAVHTWYCCDGTYYTTBAGCSAAG
	TDC-R2deg	AYTTGCCARTYYTTRWARTCNABNAC
SGD	SGD-F1deg	DKCNKCHTWYCARDDYGARGG
	SGD-R1deg	GHDSWRTARTRRTTNANNCCNABRWARTC
THAS1	THAS1-F2deg	AARJJYAARGYHGGDGAYRNRGTNG
	THAS1-R2deg	GCDGMVAANTCNANCATYTCYTG
THAS2	THAS2-F2deg	TDRBHDTNARRDTBVYNAARGCNTTHGG
	THAS2-R2deg	TKHKBNGCDGCRAANTYNANCATYTC
NPF2.9	NPF-Fdeg	GGTTTCTCTTYRTVTGGAATGCTGCTTC
	NPF-Rdeg	GCTTGTCCYGGAAAATGRAATGCTTCTC

Table S4. Primers used for RACE

Primer	Sequence 5′----3′
RACE 3′	
GeneRacer™ 3′ Primer	5′-CGCTACGTAACGGCATGACAGTG-3′
*HpTHAS*R-F1	5′GAC GGA ACC CCC ACA TAT GGA GG 3′
*HpTHAS*R-F2	5′ACC TTA GCA TAG GAA GGA AAA TTG 3′
*HpTDC*R-F1	5′CTC AAT GAG TTT CAG CCC TCA TAA ATG G 3′
*HpTDC*R-F2	5′TGC TGG TCA AGG CAC TAA GTA CTA AC 3′
HpLAMT-F	5′CTT CAG ATC TCT CCC GCC AAA CAG G 3′
RACE 5′	
GeneRacer™ 5′ Primer	5′-CGACTGGAGCACGAGGACACTGA-3′
HpLAMT-R	5′GAG AGC CAG TGA AGT GCA TAA GAG C 3′

Table S5. Primers used for RT-qPCR

**Gene**	**Primer name**	**Primer Sequence**	**Amplicon size (bp)**	**Mean PCR efficiency**
Elongation factor 1-α	EF1-α-F	5′CTG AAG TTA AGT CTG TTG AGA TGC ACC 3′	200	1.77
	EF1-α-R	5′ GCC AGG GTG GTT CAT GAT GAT GAC C 3′		
Loganic Acid Methyltransferase	LAMT-F	5′CTT CAG ATC TCT CCC GCC AAA CAG G 3′	126	1.85
	LAMT-R	5′GAG AGC CAG TGA AGT GCA TAA GAG C 3′		
Tryptophan decarboxylase	TDC-F	5′GTGGATTCATGTTGATGCTGCTTATGC3′	90	1.82
	TDC-R	5′GAGTCAACTCTCTCAACCCCATCC3′		
Strictosidine β-D-glucosidase	SGD-F	5′ CAC ACA ATC AGC TTC TAG CCC ATG C 3′	96	1.81
	SGD-R	5′ACA CGA GTG TAA TCC CAA TCT TGC C 3′		
Tetrahydroalstonine synthase 1	THAS1-F	5′ GGA GTT GGT TGC CTC GTA GGA TC 3′	87	1.93
	THAS1-R	5′CGT GAA TAT TTG GGC AGG GCA GTA G 3′		
Tetrahydroalstonine synthase 2	THAS2-F	5′CTC CCC TTC TCT GTG CTG GGA TC 3′	97	1.91
	THAS2-R	5′CCA CCA AGG CCA ACC ACA CC 3′		
Nitrate/Peptide Strictosidine Transporter	NPF-F	5′CGTAATCTCTGGTGCCATCTCTCTC3′	103	2.08
	NPF-R	5′CCTAATCGTTGGAGGGGTGTTGG3′		
Strictosidine synthase	STR-F	5′GTKAGCABMRWMTATGATGACAGAGGTGT3′	131	2.04
	STR-R	5′CCTGGTAYATTYARYCCWTTCATCAAAACT3′		

Supplementary material 2.

H. patens **cloned sequences.**

>HpaLAMT (complete CDS)

ATGGCTCCAACAATGGACACCATCCCAACTGTAGACATCAAGGAAATGCC
AGAAGAAGCACACCCCATGAAAGGTGGAGATGATTTCAATAGCTATTCTC
AAAATTCTTGCTATCAGAGGGGAGTTATAGATGCCGCAAAAGCTATCATTT
TTGAAGCAGTAAATGAGAATCTAGAACTTGAGAATAACAAATCTACTTTCG
ATCCATCTAAGCCTTTTCGCATTGCTGATTTTGGTTGCTCAACCGGCCTAA
CACATTTTTAGCTATGCAAAATATCGTGGAAGCCGTGGAGCAAAAGCACA
AATCTCTACAAGAAAAAACCAACCCAGAATTCCACGTATTTTTCAATGACC
ATGTCAACAACGATTTCAACATTCTCTTCAGATCTCTCCCGCCAAACAGGG
ATTACTTTGCTGCTGGGGTGCCCGGATCGTTTTACACGAGGGTGTTACCTAG
GGCCTGTTTGCATTTTGCTCATTGCTCTTATGCACTTCACTGGCTCTCTAAGG
TTCCTAAGGAGGTTCAAGACAAGAATTCTCCCGCCTTCAACAAGGGGAGG
ATTCATTACACCGGGACTGAAAAGCATGTTGTGAAAGCTTACTTTGGTCAG
TTCCAAAAGGATATGGACGCATTTCTGAAAGCTAGAGCTCAAGAAATTGTG
GGAGGAGGGTTGATGGTGATTCAAATTCCTGGTCTTCCTAGTGGTGAGGTC
CTCTTCTCTAGGACTGGTGCTGGAATGCTTCATGCTCTTCTGGGCTCGTCCTT
GATGGAACTCGTAACTTTGGGAGTAATAAGTGAAGAAAGTGTGGATTCCTT
CAACTTGCCTCAATACCATCCATCAATTGAGGAACTAGAAATGGTGATAGA
GATGAACAAAAGTTTTAGCCTTGAAAAAATTGGAGCATTGAATCATCCTAT
GAAGAACTTGCCATTTGATGTTCAAATGACTTCGCTACAAGTAAGAGCTAT
CATGGAGGGGATTCTAACCGACCATTTTGGAGACAAAATCTTGGACCAATT
GTTCGAAATCTACACCAAGAAACTGCAAGAGAATTACAATGTTTTTGACAA
GGAAATCCGAAGAGATGCGGATTTGTTCCTTGTTCTAAAACGCAAGTGCAA
TTGA

>HpaTDC (partial)

GAGACATTCCCTGTCTTTAGCCAAGTTGAGCCCGGATATCTCCGTACCTCTC
AGCCCGAAACCGCCCCTTATCTGCCCGAAAAATTTGATGACATTTTGCATG
ATATTGAAAAAGATATAATCCCTGGAATGACCAACTGGCTAAGCCCTAATT
TTTATGCATATTTTCCAGCTACTGTTAGCTCAGCTGCTTTTCTTGGAGAAATG
TTGTGCACCGGTTTCAACTCTGTAGGGTTCAACTGGCTAGCCTCCCCGGCA
GCGACCGAGCTCGAGATGGTGGTGATGGATTGGCTGGCTCACATGCTTAAG
CTCCCCAAGTCCTTTATGTTCTCAGGCACTGGTGGTGGTGTTCTCCAGGGAA
CCACCAGCGAGTCTATTCTTTGCACCCTCATCGCCGCCCGCGACCGGGCTTT
CGAGGAAATCGGCGTGGAGAATATTGGGAAGCTTGTTGTCTATGGATCCGA

TCAGACACATTCATTTTTCGTCAAAACTTGCAAACTGGCAGGTATTTTCCCT
TGCAACATAAAAATTATCCCTACTACTGTTGAGGCCAACTTTTCCATGTCCC
CCGGTGCTCTACGTAAACAAATCGAAGCTGACGTGGCAGATGGGCTGGTC
CCACTTTTTGTATGTGCTACTGTGGGAACCACATCCACCACTGCCGTTGACT
CAGTGAGTCAGATAGCTGATGTAGCAAATGAATTCAAGATGTGGATTCATG
TTGATGCTGCTTATGCTGGTAGTGCATGCATATGTCCTGAGTTTAGGCAGTA
CTTGGATGGGGTTGAGAGAGTTGACTCAATGAGTTTCAGCCCTCATAAATG
GCTACTTTGTTATTTGGATTGTTGTTGCTTGTGGGTCCAGAGACCTGACTTGC
TGGTCAAGGCACTAAGTACTAACCCTGAATACTTGAAAAATAAAAGAAGT
GAGCTTGGCTCTGTCATGGACTACAAAGACTGGCAAATTGGTACGGGAAG
ACGATTTAGAGCACTCCGATTATGGCTTATTATGCGCAGTTACGGCCTTAA
AAATCTCCAAAGCCACATCCGGTCCGACGTCCAAATGGCCAAAATGTTCG
AAGGGTTCGTGAAATCTGATCCACGGTTTGAAATGATTGTACCGCGTGCAT
TTTCACTTGTGTGTTTTAGGCTTAATCCAAGGAGATTGGGTGAAAGTCATGC
AATTGATTTGGAGCTCTTGAACAAGAAGCTACTCGAGTCGATCAACTCAAC
TGGGCGAGTTTACATGACTCACACCAAGGTTGGTGGGGTATACCTGTTGAG
ATTTGCAGTGGGGGCCTCGTTAACAGAGGATCGCCATGTGTGTGCCGCCTG
GGAGTTGATAAAAGAATGCACCGATAATTTGCTTAAAGAAAATAATTAG

>HpaSGD (partial)

GCGGCCTATCAATATGAAGGTGGAGCAAGTGAAGGCGGTAAAGGTCCCAG
TATATGGGATACTTTCACCCATAGACGGCCTGGTTTGATAATGGGTGGAGG
CAATGGAAACCTGGCCATTGATTCATATCATCAGTACAAGGAAGATGTCA
AGATGTTGAAGAACATGGGGTTGGATGCCTATCGGTTTTCAATATCATGGT
CAAGAGTCCTGCCAGGTGGGAATTTAAATGGTGGTGTAAATAAGGAAGGA
GTTGACTACTACAACAATCTCATAGATGAGCTCCTAGACAATGGTATCGAG
CCATATATAACTCTATTTCATTGGGATGTTCCCCAAGCCTTGGAAGATAAAT
ATGGTGGTTTTTTAAGTCCAAAAATTGTGGACGACTTCGTGGAGTACGTAG
AGCTTTGCTTTTGGGAATTCGGAGATCGAGTGAAGAACTGGATAACACTGA
ATGAACCCTGGAGCTTTAGCACTGGTGGATATGTAAACGGTACCTTTGCAC
CCGGCCGAGGTGCCTCCGCCTCTGAGCAAGTAAATCACGATCACATTGTAC
CAAGCAGATGTTCTGCACATGTACGACAATGTATTTCTAGCAATGGAAATC
CAGGCACGGAGCCTTATTTGGTCACACACAATCAGCTTCTAGCCCATGCTG
CTGCTGTAAAACTATACAAAAGCAAATTTCAGAAACCACAAGAAGGCAAG
ATTGGGATTACACTCGTGTCTCAATGGTTGGAACCATTGGATGAAAACAAT
GATGCCGATGTCCAAGCGTCCAAAAGAGCTCTCGATTTCATGTTAGGATGG
TTAATGCAGCCACTTAA

>HpaNPF2.9 (partial)

GGCTCAAGCTTTTGTGAAACTCCATCACCTTTGCAATATACAAGCCTATTTT
TGGCTATAGCTCTGGCAGCTTTAGGGTGTGCAGGTACTAGTTTCACTGTCGG
AACAATGGGAGCAGATCAGTTGGATAATCCTGAGCATCAAGAGAATTTCTT
TAACTGGTTTCTTTTCATCTGGGATGCTGCTTCACTGATTAGTGGTACCGTA
ATTGTTTATGTTCAAGATAATGTGAGTTGGGGATTGGGGTATGGCCTATGTG
GTGCCGCTAATCTGCTAGGATTAATCAGTTTTCTGCTGGGAAGGCACTACT
ATCGCTATGTTCAACCACAAGGGAGTCCGTTCAAGGATGTAGCTCGCGTAG
TCTTTGCAGCATTCTCTAAGAGGAAGCAATTTTTGTCAAAAACAAGAGAAG
ATTATTATAGTCCATTACATAACGAAGCCGATGACCAACATGACGGTGTCA
ATTATTTGGCAGCAGCAGCCTCACAACCAAAAGAAACATTCAAGTTTCTGA
ACCATGCAGCCTTGATAACCCAAGCAGACATCCAATCAGATGGATCAATC
AGGCAATCATGGAAGTTGTGCACGGTACAACATGTGGAAGATCTTAAAAC
CTTAATTAGAATTGCCCCAATTTGGGCAACTGTTATTTTCATAACCACCCCA
ATGAGTATGCTAGCCGCCTTCACACTCCTTCAGGCTCTAAAAATGGACACT
GGCATTGTATCCAATTTCAAATTCCCAGTAGGTTCTCTGGTAGTCTTTTGCG
TAATCTCTGGTGCCATCTCTCTCACCATATTAGACAGATTGATATTCCCTTT
ATGGCAAAAAATATTCGGCAAATTTCCAACACCCCTCCAACGATTAGGCA
CAGGTCATGTCATAAATGTGCTTAGCATAGTCGTTGCAGCCTTGGTGGAAT
CAAAGAGGCTGCAATTAGCTCGAGCCCACAATGTTCAAGATTCGAGAATTT
CCATCATGCCAATGTCTGTTATGTGGTTAGTACCTCAGTTGGCACTTATGGG
AATAGGAGAAGCATTCCATTTTCCAGGACAAGC

>HpaTHAS1 (partial)

AAAGTTAAAGTTGGGGACGAGGTGGGAGTTGGTTGCCTCGTAGGATCATGT
CGAAATTGTAACAGGTGCAACGAAGACCTTGAAAACTACTGCCCTGCCCA
AATATTCACGTATGGCTCCCCAGACACCGACGGAACCCCCACATATGGAG
GCTATTCAGATATCATGGTTGTGGACGAGCACTTCGTTGTTCGTTGGCCTGA
AAACTTCCCTCTTGATGCCGGTGCACCCCTTCTCTGTGCTGGAATCACTACA
TATAGCCCGTTGAGATATTTTGGACTTGACAAACCTGGAATTCATATTGGTG
TGGTTGGCCTTGGTGGGCTTGGTCATGTGGCCGTGAAATTCGCCAAGGCAT
TTGGGGCAAAGGTGACCTTGATCAGCACTTCCGCTAGTAAAAAACAGGAT
GCCATCGAAAAACTAGGGGCTGATTCATTTATACTTAGTAATGACCCTAAG
GAAATGGAGGCTGCATTTGGAACTATGGATGGTATTATTGATACTGTCTCT
GCTGTTCATTCAATTCTGCCTTTGATTGATTTGTTAGCATTTGATGGGAAGCT
TGTTGAGGTTGGTATACCAGCAAAGCCACTTGAACTGCCCATATATAACCT
TAGCATAGGAAGGAAAATTGTGGGAGCGACTACTATTGGAGGCTTGAAGG
AAACGCAAGAAATGATTGATTTTGCAGTAAAGCACAATATAGTACCTGAT

GTGGAGATCATCCCAATTGATTATGTGAACACCGCAATGGAGCGCCTTGAG
AGGGGTGAAGTCAAGTACCGATTTGTGATAGATGTGGGGAATACACTAAA
GTCTACTTAG

>HpaTHAS2 (partial)

GGTAAAGTTGGAGATGTGGTAGGAGTGGGTTGCCTAGTTGGATCATGTCGC
AACTGTGACATGTGTTCCAAAGATCTCGAGAACTACTGTCCTGGGCAGATA
CTCACATACAGCGCCGCTTATACCGATGGTACAATCACATATGGAGGCTAC
TCTGATCTTATGGTTGCTGATGAGCACTTTGTGATCCGCTGGCCCGAGAACT
TTCCTCTTGATGCTGGTCGTCCCCTTCTCTGTGCTGGGATCACTACTTACAGC
CCCTTGAGATACTTTGGACTTGATAAGCCTGGAACCCATGTTGGTGTGGTTG
GCCTTGGTGGGCTTGGTCACGTTGCTGTGAAATTTGCCAAGGCTTTTGGGGC
AAAGGTGACTGTGATCAGTACCTCTGAGGGGAAAAAGAAGGAGGCCATTG
AAAAACTTGGAGCAGACGCGTTCTTAAATAGCCGGGACCCTGAGCAGATG
CAGGCTGCACGTGGTACAATAGATGGTATTATTGATACTGTTTCCGCAACA
CATCCTGTTATTCCATTGATCAATTTGGTGAAGTCTCATGGGAAGATAATAC
TGGRRGGTGCAGGCGAAAAGCCACTTGAGGTTCCYGCATTTCCGCTTTTGA
TCGGGAGGAAGACGCTTACTGGGAGTGCTATTGGCAGGTCTGAAGGAAAC
TCAGGAGATGCTGGACTTCGCAGC

Supplementary material 3.

Accesion numbers of amino acid sequences used for phylogenetic analysis.

Loganic acid O-methyltransferase (LAMT)

Ophiorrhiza pumila (OpuLAMT) -QWX38535.1
Mitragyna speciosa (MspLAMT)-[37]
Mitragyna speciosa (MspLAMT2)- [37]
Catharanthus roseus (CroLAMT)- AGX93063.1
Uncaria tomentosa (UtoLAMT)- QDD67562.1

Tryptophan decarboxylase (TDC)

Catharanthus roseus (CroTDC)- AYA72254.1
Rauwolfia tetraphylla (RteTDC)-WMM65729.1
Rauwolfia verticillata (RveTDC)- ABP96805.1
Vinca minor (VmiTDC)- AEY82397.1
Tabernaemontana elegans (TelTDC)- AEY82396.1
Ophiorrhiza pumila (OpuTDC2)- QLQ34389.1
Mitragyna speciosa (MspTDC)- AEQ01059.1
Ophiorrhiza prostrata (OprTDC)- ABU40982.1
Ophiorrhiza pumila (OpuTDC)- BAC41515.1
Uncaria tomentosa (UtoTDC)- QDD67561.1
Uncaria guianesis (UguTDC)- ASY03221.1
Camptotheca acuminata (CacTDC)- AAB39708.1
Camptotheca acuminata (CacTDC2)-AAB39709.1
Uncaria rhynchophylla (UryTDC)- [56]

Tetrahydroalstonine synthase (THAS)

Uncaria tomentosa (UtoHYS)-QDD67564.1
Uncaria tomentosa (UtoTHAS)- QDD67563.1
Catharanthus roseus (CroHYS)-ANQ45225.1
Catharanthus roseus (CroTHAS1)-KM524258.1
Catharanthus roseus (CroTHAS2)- ANQ45223.1
Catharanthus roseus (CroTHAS3)- ANQ45222
Catharanthus roseus (CroTHAS4)- KU865324
Rauvolfia tetraphylla (RteTHAS)-MH010799.1
Rauvolfia tetraphylla (Rtiso1THAS)-ASR92154.1
Mitragyna speciosa (MspTHAS)-WNF20697.1

Rauvolfia tetraphylla (RteTHAS3)- OR514625
Rauvolfia tetraphylla (RteTHAS4A)- OR514623
Rauvolfia tetraphylla (RteTHAS4B)- OR514624
Rauvolfia tetraphylla (RteTHAS5)- OR514622
Rauvolfia tetraphylla (RteYOS)- OR514626
Rauvolfia tetraphylla (RteAMS)- OR514628
Rauvolfia tetraphylla (MSTRG.5534)-OR514630
Rauvolfia tetraphylla (MSTRG.5531)-OR514631
Rauvolfia tetraphylla (MSTRG.5530)-OR514632.

Strictosidine beta-glucosidase (SGD)

Mitragyna speciosa (MspSGD1) - WNF20694.1
Rauwolfia verticillata (RveSGD)-AFI71457.1
Rauwolfia serpentina (RseSGD)- CAC83098.1
Catharanthus roseus (CroSGD)-AAF28800.1
Catharanthus roseus (CroSGDs)- AVH80943.1
Uncaria tomentosa (UtoSGD)- AFN69080.1

Amino acid sequences alignment

Loganic acid O-methyltransferase (LAMT)

```
Consensus  ---------MAPTXDXX-----PXXDXKEMPEEAHPMKGGDDXNSYSQNSCYQKGVIDAAKXVIXEAVXE

CroLAMT    ---------MVATIDSIEM------PALPTAVEAHPMKGGDDSHSYSQNSCYQKGVIDAAKAVIVEAVNE   55
OpuLAMT    ---------MAPTMDNNTTTITTVEIIKEMPEEAHPMKGGDDLNSYSQNSCYQKGVIEAAKAVIVEAVHE   61
HpaLAMT    ---------MAPTMDTI-----PTVDIKEMPEEAHPMKGGDDFNSYSQNSCYQRGVIDAAKAIIFEAVNE   56
MspLAMT2   MAPTVKTPTPIPTPNPN-----PSLDIKVMPEEAHPMKGGDDLNSYSQNSCYQKGVIDAAKSVIIEAVTE   65
MspLAMT    ---------MAPTLDTI-----PTVDVKEMPEEAHPMKGGDDFNSYSQNSCYQRGVIDAAKVVIVEAVNE   56
UtoLAMT    ---------MAPTMDTV-----PTVDVKEMPEEAHPMNGGDDLNSYSQNSCYQKGVIDAAKTVILEAVTE   56

Consensus  KLDLENNX-XXFDPSKPFRIADFGCSTGPNTXFAMQNIVEAVXXKYKSLXXKXQPEFHVFFNDHVNNDFN

CroLAMT    KLDLENN--PIFDPIKPFRIADFGCSTGPNTFHAMQNIVESVETKYKSLQK--TPEFHVFFNDHVNNDFN   121
OpuLAMT    KMDLENNIITTFDPSKPFRIADFGCSTGPNTFFAMQNIVEVVDNRYKSLLKNQQVEFHVYFNDHANNDFN   131
HpaLAMT    NLELENNK-STFDPSKPFRIADFGCSTGPNTFFAMQNIVEAVEQKHKSLQEKTNPEFHVFFNDHVNNDFN   125
MspLAMT2   KLDLENNI-TTFDPAKPFRIADFGCSTGPNTYFAMQNVVEAVELKYKSL-KKDQPEFHVFFNDHVNNDFN   133
MspLAMT    KLDLKNN--ANFDPSKPFHIADFGCSTGPNTYFAVQNIVEAVDQKHKSLHEKIQPEFHVFFNDHVNNDFN   124
UtoLAMT    KLDLENN--ANFDPSKPFRIADFGCSTGPNTYFAMQNIVEAVDQKYKSQLGKSQPEFHVFFNDHVNNDFN   124

Consensus  ILFRSLPQNRDYFAAGVPGSFYTRVFPKASLHFAHCSYALHWLSKVPKEIQDKNSPAXNKGRIHYTGTEK

CroLAMT    VLFRSLPPNREFFAAGVPGSFYTRVFPKNSIHFAHCSYALHWLSKVPKEIQDKNSLAYNKGRIHYTGTEK   191
OpuLAMT    MLFRSLPQNRDYFAAGAPGSFYSRVFPKASLHFAHCSYALHWLSKVPKEIQDKNSPAYNKGRIHYTGTEK   201
HpaLAMT    ILFRSLPPNRDYFAAGVPGSFYTRVLPRACLHFAHCSYALHWLSKVPKEVQDKNSPAFNKGRIHYTGTEK   195
MspLAMT2   ILFRSLPQNRDYFAAGVPGSFYTRVFPKASLHFAHCSYALHWLSKVPKEIQDKNSPAFNKGGIHYTGTEK   203
MspLAMT    ILFRSLPQDRDYFAAGVPGSFYTRVFPKASVHFAHCSYALHWLSKVPKEIQDKNSPAFNRGRIHYTGTEK   194
UtoLAMT    ILFRSLPQDREYFAAGVPGSFYTRVFPKASLHFAHCSYALHWLSKVPNEIQDKNSPAYNKGRIHYTGTEK   194

Consensus  HVVKAYFXQFQKDMXSFLKARAQEIVGGGLMVIQIPGLPSGEVLFSRTGAGMLHALLGSSLMELVNLGXI

CroLAMT    HVVKAYFGQFQRDFEGFLKARAQEIVVGGLMVIQIPGLPSGEVLFSRTGAGLLHFLLGTSLMELVNKGII   261
OpuLAMT    QVVKAYFSQFQKDLDSFLKARAQEIVGGGLMVIQIPGLPSGEVLFSRTGAGMLHALLGSSLMDLVNMGII   271
HpaLAMT    HVVKAYFGQFQKDMDAFLKARAQEIVGGGLMVIQIPGLPSGEVLFSRTGAGMLHALLGSSLMELVTLGVI   265
MspLAMT2   HVVKAYFGQFQKDMDSFLKARALEIVGGGLMVIQIPGLPSGEVLFSRTGAGKLHALLGASLMELVNLGVI   273
MspLAMT    HVVKAYFSQFQKDMGSFLKARAQEIVGGGLVVIQIPGLPSGEVLFSRTGAGMLHALLGS-LMDLVNLGII   263
UtoLAMT    HVVKAYFSQFQKDMGSFLKARAQEIVGGGLMVIQIPGLPSGEVLFSRTGAGMLHALLGSSLMELVNLGVI   264

Consensus  XEESVDSFNLPQYHPSIEELEMVIEMNXSFTLEKXGALNHPMKNLPFDVQMTSLQVRAIMEGILTDHFGX

CroLAMT    NEESVDSFNLPQYHPSVEDLEMVIEMNDCFTIERVGTLPHPMKNLPFDVQRTSLQVRAIMECILTEHFGE   331
OpuLAMT    SEESVDSFNLPQYHPSIEELEMLIEMNGSFTLEKIGALNHPMKNLAFDAQMTSLQVRAIMEGILTKQFGD   341
HpaLAMT    SEESVDSFNLPQYHPSIEELEMVIEMNKSFSLEKIGALNHPMKNLPFDVQMTSLQVRAIMEGILTDHFGD   335
MspLAMT2   SEKSVDSFNLPQYHPSIEELEMVIEMNNSFTLEKIGALNHPMKSLPFDVQMTSLQVRAIMEGILTDHFGE   343
MspLAMT    TEESVDSFNLPQYHPSIEELEMVIEMNNSFTIEKVGALNHPMKNLPFDVQMTSLQIRAVMEGILSDHFGG   333
UtoLAMT    TEESVDSFNLPQYHPSIEEMEMVIEMNNNFTLEKAGALNHPMKNLPFDVQMTSLQVRAVMEGILTDHFGE   334

Consensus  KILDQLFEIYTXKLQENYNVFDKEIRRDADLFLVLKRKXN

CroLAMT    NILDPLFEIYTKNLQENFHVFDKEIRKDADLYLVLKRKGN   371
OpuLAMT    KILDQLFEIYTNKLQENYIVFDKEIRKDADLFLILKRKIN   381
HpaLAMT    KILDQLFEIYTMKLQENYNVFDKEIRRDADLFLVLKRKCN   375
MspLAMT2   KILDQLFEIYTKKLQENYNVFDKEIRRDADLFLVLKRKSK   383
MspLAMT    KILDQLFEIYTTKLQENYNVFDKEIRRDADLFLVLKRKSN   373
UtoLAMT    NILDPLFEIYTKKLQENYNVFDKDIRRDADLFLVLKRKSN   374
```

Tryptophan decarboxylase (TDC)

```
Consensus  MGSIDXXXXX-XXSXSXVAXFKPLXXEEFRKQAHXMVDFIADYYKNXEXYPVLSQVEPGYLRXRLPETAP

CroTDC     MGSIDSTNVA--MSNSPVGEFKPLEAEEFRKQAHRMVDFIADYYKNVETYPVLSEVEPGYLRKRIPETAP  68
VmiTDC     MGSIDSTNDVALSNGSSVGEFKPLEAEEFRKQAHCMVDFIADYYKNVESYPVLSQVEPGYLRERLPETAP  70
TelTDC     MGSIDSTNVA--PSTPSIAEFKPLDAEEFRKEAHRMVDFIADYYKNVENYPVLSQVEPGYLRERLPENAP  68
RveTDC     MGSIDSTDVA--ISASPVAEFKPLEAEEFRKQAHRMVDFIADYYKNVESYPVLSQVEPGYLRERLPETPP  68
RteTDC     MGSIDSTDVA--FSASAVAEFKPLEAEEFRKQAHRMVDFIADYYKNVESYPVLSQVEPGYLRERLPESPP  68
CacTDC     MGSLDSNYD--TESPASVGQFNPLDPEEFRKQAHCIVDFIADYYKNIESYPVLSQVDPGYRHSRLGKNAP  68
CacTDC2    MGSIDSNYD--TES---AGQCRPLEPEEFRKQAHQMVDFIADYYKNIESYPVLSQVEPGYLQSRLPETAP  65
OpuTDC     MGSISENC---DDSISLAAPFRPLEPEEFRKQAHVMVDFIADYYKNIENYPVLSQVEPGYLKNRLPETAP  67
OprTDC     MGSISENC---DDSISLAAPFRPLEPEEFRKQAHIMVDFIADYYKNIENYPVLSQVEPGYLKNRLPETAP  67
HpaTDC     -----------------------------------------------ETFPVFSQVEPGYLRTSQPETAP  23
OpuTDC2    MGSIDANNINGAYPSSPVAPFKPLDPEEFRKQAHVMVDFIADYYKNIEKYPVLSQVEPGYLRTRLPETAP  70
UtoTDC     ------------------------------------------------RQPVLSQVEPGYLRKHLPETAP  22
MspTDC     MGSIDTSD-GDAYANSAVAPFKPLDPDEFRKQAHRMVDFIADYYKNIENYPVLSQVEPGYLRTQLSQTAP  69
UryTDC     MGSIDTND-DAAYANSAVAPFKPLDPEEFRKQAHFMVDLIADYYKNIENYPVLSQVEPGYLRARLPETAP  69
UguTDC     ----------------------------------------------------------------------


Consensus  YLPEXFEXILXDIQKDIIPGMTNWLSPNFFAXFPATVSSAAFLGEMLCTXFNSVGFNWLASPAATELEMV

CroTDC     YLPEPLDDIMKDIQKDIIPGMTNWMSPNFYAFFPATVSSAAFLGEMLSTALNSVGFTWVSSPAATELEMI  13
VmiTDC     YLPESLDKIMSDIQKDIIPGMTHWMSPNFYAFFPATVSSAAFLGEMLSTALNSVGFTWVSSPAATELEMI  14
TelTDC     YLPESLDMIINDIQKDIMPGMTNWMSPNFYAFFPATVSSAAFLGEMLSTALNSVGFTWISSPAATELEMI  13
RveTDC     YLPDSLDKIIDDIQKDIIPGMTNWMSPNFYAFFPATVSSAAFLGEMLSTALNSVGFTWVSSPAATELEMI  13
RteTDC     HLPESLDKIIDEIQKDIIPGMTNWMSPNFYAFFPATVSSAAFLGEMLSTALNSVGFTWVSSPAATELEMI  13
CacTDC     YRSEPFESILKDVQKDIIPGMTHWMSPNFFAHFPATVSSAAFVGEMLCTCFNSVGFNWLASPAATELEMV  13
CacTDC2    YRPEPFESILKDVHKDIIPGVTHWLSPNFFAYFPATVSSAAFVGEMLCTCFNAVGFNWLASPAELELEMV  13
OpuTDC     HLPESFETILKDIKKDIVPGMTNWLSPNFFAYFPATVSSAAFVGEMLCTGFNSVGFNWLASPASTELEMV  13
OprTDC     HLPESFETILKDIKKDIVPGMTNWLSPNFFAYFPATVSSAAFVGEMLCTGFNSVGFTWLASPASTELEMV  13
HpaTDC     YLPEKFDDILHDIEKDIIPGMTNWLSPNFYAYFPATVSSAAFLGEMLCTGFNSVGFNWLASPAATELEMV  93
OpuTDC2    YLPEPFENILQDIQKDIIPGMTNWLSPNFFAYFPATVSSAAFLGEMLCTGFNSVGFNWLASPAATELEMV  14
UtoTDC     YLPEPFENILHDIQKDIIPGMTNWLSPNFFGFFPATVSSAGFLGEMLCTGFNSVGFNWLASPAAAELEMV  92
MspTDC     YLPEPFENILQDIQKDIIPGMTNWLSPNFFAFFPATVSSAAFLGEMLCTGFNSVGFNWLASPAATELEMV  13
UryTDC     YLPEPFENIMQDIHKDIIPGMTNWLSPNFFAYFPATVSSAAFLGEMLCTGFNSVGFNWLASPAATELEMI  13
UguTDC     ----------------------------------------------------------------------


Consensus  VMDWLAXMLKLPKSFMFSGTGGGVIQGTTSEAILCTLIAARDRALEKXGVXNIGKLVVYGSDQTHSXFXK

CroTDC     VMDWLAQILKLPKSFMFSGTGGGVIQNTTSESILCTIIAARERALEKLGPDSIGKLVCYGSDQTHTMFPK  20
VmiTDC     VMDWLAKMLKLPECFMFSGTGGGVIQNTTSESILCTIIAARERVLENLGPNSIGKLVCYGSDQTHTMFPK  21
TelTDC     VMDWFAQMLKLPKSFMFSGTGGGVIQNTTSESILCTIIAARERALDKLGQDNIGKLVCYGSDQTHTMFPK  20
RveTDC     VMDWLAQMLKLPKSFMFSGTGGGVIQNTTSESILCTIIAARERALEELGVDSIGKLVCYGSDQTHTMFPK  20
RteTDC     VMDWLAQMLKLPKSFMFSGTGGGVIQNTTSESILCTIIAARERALEELGVDSIGKLVCYGSDQTHTMFPK  20
CacTDC     VIDWLANMLKLPKSFMFSGTGGGVLQGTTSEAILCTLIAASPMHFEIVGVKTSTSFVVYGSDQTHSTYAK  20
CacTDC2    VMDWLASMLKLPNSFTFLGTGGGVIQGTTSEAILCTLIAARDRALESIGVDSIHKLVVYGSDQTHSTYAK  20
OpuTDC     VIDWLANMLKLPKSFMFHGTGGGVIQGTTSEAILCTLIAARDGALEKIGMENVGKLVVYGSDQTHSFFQK  20
OprTDC     VIDWLANMLKLPKSFMFHGTGGGVIQGTTSEAILCTLIAARDGALEKIGMENVGKLVVYGSDQTHSFFQK  20
HpaTDC     VMDWLAHMLKLPKSFMFSGTGGGVLQGTTSESILCTLIAARDRAFEEIGVENIGKLVVYGSDQTHSFFVK  16
OpuTDC2    VIDWLANMLKLPNSFMFSGTGGGVLQGTTSEAILCTLIASRDRAFEKIGVENIGKLVVYASDQTHSFFHK  21
UtoTDC     VMDWLADMLKLPKSFMFSGDGGGVLQGTTSEAILCTLIAARDHVLNKIGVKNIGKLVVYGSDQTHTFFIK  16
MspTDC     VMDWLANMLKLPKSFMFSGTGGGVLQGTTSEAILCTIIAARDRAFEKIGVENIGKLVVYASDQTHSFFVK  20
UryTDC     VMDWMANMLDLPKSFMFSGTGGGVLQGTTSEAILCTLIAARDRAFEKFGADNIGKLVVYASDQTHSFFVK  20
UguTDC     ----------------------------------------------------------------------
```

```
Consensus  TCKLAGIFPCNIRXIPTTXEXNFSMSPXALRXQIEADVAXGLVPLFLCATVGTTSTTAIDPVSXLAXVAN

CroTDC     TCKLAGIYPNNIRLIPTTVETDFGISPQVLRKMVEDDVAAGYVPLFLCATLGTTSTTATDPVDSLSEIAN   278
VmiTDC     TCKLAGIFPDNIRLIPTTLETDFSIDPHVLREMVKADVDAGLIPLFLCATLGTTSTTATDPVSSLSEITN   280
TelTDC     TCRLAGIFPSNIRLVPTTAETDFSIAPEVLRKMVEADVAAGLTPLFLCATLGTTSTTATDPVNALAEIAN   278
RveTDC     TCKLAGISPKNIRLIPTTAETDFGIAPEVLRGMVEADIAAGLVPLFLCATLGTTSSTATDPVDSLSEIAN   278
RteTDC     TCKLAGISPNNIRLVPTTAETDFGIAPEVLRRMVEADVAAGLVPLFLCATLGTTSSTATDPVDTLSEIAN   278
CacTDC     ACKLAGILPCNIRSIPTTADSNFSVSPLLLRRAIEADKAAGMVPLYICATVGTTSTTAIDPLSSLADVAN   278
CacTDC2    ACNLAGILPCNIRSIRTEAVANFSLSPDSLHREIEADVAAGMVPLYLCATVGTTSTTAIDSLSPLADVAN   275
OpuTDC     TCKVAGIFPCNIKLIPTTREDNFSMSPIALREQIEADVADGLVPIFLCTTVGTTSTAAIDPVSEVAKVAN   277
OprTDC     TCKVAGIFPCNIKLIPTTREDNFSMSPIALREQIEADVADGLVPIFLCATVGTTSTAAIDPVSEVAKVAN   277
HpaTDC     TCKLAGIFPCNIKIIPTTVEANFSMSPGALRKQIEADVADGLVPLFVCATVGTTSTTAVDSVSQIADVAN   233
OpuTDC2    TCKLAGIFPCNIKIIPTTSEANFSMSPHALRKQIESDVAGGLVPIFLCATIGTTSTTAIDPVSGLAKVAN   280
UtoTDC     TCKLAGIFPCNIRIIPTTAEANFSMSPVALRQQIEADAADGLVPLFLCATVGTTSTTAVDPVSQIAHVAN   232
MspTDC     TCKLAGIFPCNIRIIPTTADDNFSMSPDALRKQIEADVEDGLVPLFICATVGTTSTTAIDPVSELADVAN   279
UryTDC     TCKLAGIFPRNIRIIPTTVENYFSMSPEAVRKQIQADVENGLVPLFLCATVGTTSTTAIDSVSQLADIAN   279
UguTDC     ------------------------MSPEAVRKQIQADVKNGLIPLFLCATVGTTSTTAIDSVSQLADIAN   46

Consensus  EFNXWIHVDAAYAGSACICPEFRXYLDGIERVDSLSLSPHKWLLXYLDCCCLWVKKPXLLVKALXTNPEY

CroTDC     EFGIWIHVDAAYAGSACICPEFRHYLDGIERVDSLSLSPHKWLLAYLDCTCLWVKQPHLLLRALTTNPEY   348
VmiTDC     EFNIWMHVDAAYAGSACICPEFRHYLDGIERVDSLSLSPHKWLLAYLDSTCLWVKNPNLLLRALTTNPEY   350
TelTDC     EFDIWIHVDAAYAGSACICPEFRHYLDGIERVDSLSISPHKWFLAYLDCTCLWVKKPQLLLRALTTNPEY   348
RveTDC     EFNIWMHVDAAYAGSACICPEFMHYLDGIERVDSLSISPHKWLLAYLDCTCLWVKKPHFILRALTTNPEY   348
RteTDC     EFNIWMHVDAAYAGSACICPEFRHYLDGVERVDSLSLSPHKWLLAYLDCTCLWVKKPYLLLRALTTNPEY   348
CacTDC     DYGVWFHVDAAYAGSACICPEFRHYLDGIERADSLSLSPHKWLLSYLDCCCLWVKSPSLLVKALSTDPEY   348
CacTDC2    DYGLWFHVDAAYAGSACICPEFRHYLDGIERADSLSLSPHKWLLSYLDCCCLWVKRPSVLVKALSTDPEY   345
OpuTDC     DFNIWVHVDAAYAGSACICPEFRQYLDGIELVDSFSLSPHKWLLCFLDCCCLWLKKPHLMVKALSTNPEY   347
OprTDC     DFNIWVHVDAAYAGSACICPEFRQYLDGIELVDSISLSPHKWLLCFLDCCCLWLKKPHLMVKALSTNPEY   347
HpaTDC     EFKMWIHVDAAYAGSACICPEFRQYLDGVERVDSMSFSPHKWLLCYLDCCCLWVQRPDLLVKALSTNPEY   303
OpuTDC2    DFSVWIHVDAAYAGSACICPEFRKYLDGVELVDSLSLSPHKWLLCYLDCCCLWVKKPALMVKALSTNPEY   350
UtoTDC     EFNVWIHVDAAYAGSACICPEFRQYLDGIERADSLSLSPHKWLLCYLDCCCLWVKKPDLMVKALSTNPEY   302
MspTDC     DFNVWIHVDAAYAGSACICPEFRQYLDGIERVDSLSLSPHKWLLCYLDCCCLWVKKTDLLVKALATNPEY   349
UryTDC     EFNVWIHVDGAYAGSACICPEFRQYLEGVERVDSLSLSPHKWLLCYLDCCCLWVKKTDLLVKALATNPEY   349
UguTDC     EFNVWIHVDGAYAGSACICPEFRQYLEGVERVDSLSLSPHKWL---------------------------   89

Consensus  LKNKXSEXDXVVDFKDWQIGTGRXFRALRLWLXMRSYGVXNLQSHIRSDVXMAKMFEGFVXSDPRFEXVV

CroTDC     LKNKQSDLDKVVDFKNWQIATGRKFRSLKLWLILRSYGVVNLQSHIRSDVAMAKMFEEWVRSDSRFEIVV   418
VmiTDC     LKNKQSDLDKVVDFKNWQIATGRKFRSLKLWLILRSYGVANLQTHIRSDVAMAKMFEGFVRSDPRFEVVV   420
TelTDC     LKNKQSELDKVVDFKNWQIATGRKFRALKLWFILRSYGVANLQSHIRSDVAMAKMFEGFVRSDPRFEVVV   418
RveTDC     LKNKQSELDKVVDFKNWQIATGRKFRALKLWLILRSYGVSNLQSHIRSDVAMAKMFEDFARSDPRFEVVV   418
RteTDC     LKNKQSELDKVVDFKNWQIATGRKFRALKLWLILRSYGVENLQSHIRSDVAMAKMFEDFVRSDPRFEVVV   418
CacTDC     LKNQPSESKSVVDYKDWQVGTGRRFKALRLWFVMRSYGVANLQSHIRTDVQMAKMFEGFVKSDPRFEILV   418
CacTDC2    LKNKPSESNSVVDFKDWQVGTGRRFKALRLWFVMRSYGVANLQSHIRSDIQMAKMFEEFVNSDPRFEIVV   415
OpuTDC     LRNKRSEFDGVVDFKDWQIGTGRRFKALRLWLVMRSYGVENLKRHILSDVQMAKMFEGLVKSDPRFEIIV   417
OprTDC     LRNKRSEFDGVVDFKDWQIGTGRRFKALRLWLVMRSYGVENLKRHILSDVQMAKMFEGLVKSDPRFEIIV   417
HpaTDC     LKNKRSELGSVMDYKDWQIGTGRRFRALRLWLIMRSYGLKNLQSHIRSDVQMAKMFEGFVKSDPRFEMIV   373
OpuTDC2    LRNKRSEFDSVVDFKDWQIGTGRRFRALRLWLIMRSYGVANLQRHIRSDVEMAKMFEGFVKSDPRFEVVV   420
UtoTDC     LRNKRSEFDSVMDYKDWQV---------------------------------------------------   321
MspTDC     LRNKRSEFDSVVDYKDWQIGTGKRFRALRLWLVMRCYRVANLQSHIRSDVQMAKMFEGFVKSDPRFEMIV   419
UryTDC     LRNKRSEFDSVVDYKDWQIGTGKKFRSLRLWLVMRCYGVANLQSHIRSDVQMAKMFEGFVRSDPRFEIVV   419
UguTDC     ----------------------------------------------------------------------   89
```

```
Consensus  PRXFSLVCFRLNPXX-GXXXXXDXEXLNKKLLDXXNSTGRVYMTHTKVGGIYMLRFAVGXXLTEEXHVXA

CroTDC     PRNFSLVCFRLKPDVSS----LHVEEVNKKLLDMLNSTGRVYMTHTIVGGIYMLRLAVGSSLTEEHHVRR  484
VmiTDC     PRNFSLVCFRLKPLP-G----SDVEILNKKLNDMLNSTGRVYMTHTIVGGIYMLRLAVGSSLTEEHHVRA  485
TelTDC     PRAFSLVCFRLKPFP-G----SDVEALNKKLLDKLNSTGRVYMTHTIVGGIYMLRLAVGSSLTEERHVRA  483
RveTDC     PRNFSLVCFRLKALP-G----SDVEALNKKLLDMLNSTGRVYMTHTIVGGIYMLRLAVGSSLTEEHHVRA  483
RteTDC     PRNFSLVCFRLKALP-G----SDVETLNKKLLDMLNSTGRVYMTHTIVGGIYMLRLAVGSSLTEEHHVRS  483
CacTDC     PRVFSLVCFRLNPIS--GSDPTGTEALNRKLLDWVNSTGRVYMTHTKVGGIYMLRFAVGATLTEKRHVSS  486
CacTDC2    PRVFSLVCFRLNPFS--KSDPCNTELLNRKLLEWVNSTGQVYITHTKVGGVYMLRFAVGATLTEEHHVSA  483
OpuTDC     PRAFALVCFRLNPGK-GYDDEIDKEILNKELLDLINSTGRAYMTHTKAGGIYMLRFAVGTTLTEEHHVYA  486
OprTDC     PRAFALVCFRLNPGK-GYDDEIDKEILNKELLDLINSTGRAYMTHTKAGGIYMLRFAVGTTLTEEHHVYA  486
HpaTDC     PRAFSLVCFRLNPRRLGESHAIDLELLNKKLLESINSTGRVYMTHTKVGGVYLLRFAVGASLTEDRHVCA  443
OpuTDC2    PRTFSLVCFRLNPFG-RSNNATYVELLNEKLLDLVNSTGRAYMTHTKVGGVYMLRFAVGATLTEERHVNA  489
UtoTDC     ----------------------------------------------------------------------  321
MspTDC     PRAFSLVCFRLNPSG-GSNEA-DLELLNKKLLDRVNSTGRTYMTHTKAGEVYLLRFAVGATLTEDRHVYA  487
UryTDC     PRAFSLVCFRLNPSE-GSSEA-HLELLNKKLLERVNSTGRAYMTHTKAGEVYVLRFAVGATLTEDRHVYA  487
UguTDC     ----------------------------------------------------------------------  89

Consensus  AWELIKEXXDXLLKEX----------

CroTDC     VWDLIQKLTDDLLKEA----------  500
VmiTDC     VWELIKKLADDLLKEA----------  501
TelTDC     VWELIKELANDSLKEA----------  499
RveTDC     VWELIKELANDLLKEA----------  499
RteTDC     VWELIKELTSDLLKEA----------  499
CacTDC     AWKLIKEGADVLLKED----------  502
CacTDC2    AWKLIREGADALLCS-----------  498
OpuTDC     AWELIKECTDASLTKTNIIE------  506
OprTDC     AWELIKECTDASLTKTNIIESRANPQ  512
HpaTDC     AWELIKECTDNLLKENN*--------  461
OpuTDC2    AWELIKECTAALLKENHQCIDI----  511
UtoTDC     --------------------------  321
MspTDC     AWELIKQCADAVLKENVLD-------  506
UryTDC     AWELIKACADAVIKENLLD*------  507
UguTDC     --------------------------  89
```

Strictosidine beta-glucosidase (SGD)

Consensus	MxxxxxxxLVVAIxPxxxxxxxxx--------xxxxxPxxxxKxxVHRRDFPxDFIFGAGGSAYQCEGAY	
UtoSGD	----------------------------------MSTPATKFSGTVSRSDFPEGFLFGSASSAFQYEGAH	36
HpaSGD	---AAYQYEGGA	9
MspSGD1	-----------------------------MEAQRTATVVSNDASKINRGDFAEDFIFGAASSAYQTEGGA	41
CroshSGD	MGSKDDQSLVVAISPAAEPNGNHSVPIPFAYPSIPIQPRKHNKPIVHRRDFPSDFILGAGGSAYQCEGAY	70
CroSGD	MGSKDDQSLVVAISPAAEPNGNHSVPIPFAYPSIPIQPRKHNKPIVHRRDFPSDFILGAGGSAYQCEGAY	70
RseSGD	MDNTQAEPLVVAIVPKPNASTEHT--------NSHLIPVTRSKIVVHRRDFPQDFIFGAGGSAYQCEGAY	62
RveSGD	MESNQGEPLVVAIVPKPNASTEQK--------NSHLIPATRSKIVVHRRDFPQDFVFGAGGSAYQCEGAY	62

Consensus	NEGNRGPSIWDTFTxRxPAKIxDGSNGNQAINSYHxYKEDIKIMKQxGLExYRFSISWSRVLPGGNLx--	
UtoSGD	NVDGRLPSIWDTFLVETHPDIV-AANGLDAVEFYYRYKEDIKAMKDIGLDTFRFSLSWPRILPNGRRTRG	105
HpaSGD	SEGGKGPSIWDTFTHRRPGLIMGGGNGNLAIDSYHQYKEDVKMLKNMGLDAYRFSISWSRVLPGGNLN--	77
MspSGD1	SEGGRGPSIWDTFTQRRPGMIKEGGNGNMAVDSYHQYKEDVKILKNMGLDAYRFSISWSRVLPGGNLN--	109
CroshSGD	NEGNRGPSIWDTFTNRYPAKIADGSNGNQAINSYNLYKEDIKIMKQTGLESYRFSISWSRVLPGGNLS--	138
CroSGD	NEGNRGPSIWDTFTNRYPAKIADGSNGNQAINSYNLYKEDIKIMKQTGLESYRFSISWSRVLPGGNLS--	138
RseSGD	NEGNRGPSIWDTFTQRSPAKISDGSNGNQAINCYHMYKEDIKIMKQTGLESYRFSISWSRVLPGGRLA--	130
RveSGD	NEGNRGPSIWDTFTQRTPAKISDGSNGNQAINCYHMYKEDIKIMKQAGLEAYRFSISWSRVLPGGRLA--	130

Consensus	-----xGVNKDGVKFYHDFIDELLANGIKPxxTLFHWDLPQALEDEYGGFLSxRIVDDFxEYAEFCFWEF	
UtoSGD	PNNEEQGVNKLAIDFYNKVINLLENGIEPSVTLFHWDVPQALETEYLGFLSEKSVEDFVDYADLCFREF	175
HpaSGD	-----GGVNKEGVDYYNNLIDELLDNGIEPYITLFHWDVPQALEDKYGGFLSPKIVDDFVEYVELCFWEF	142
MspSGD1	-----AGVNKEGINYYNNLIDELLANGIEPYVTLFHWDVPQALEDKYGGFLSPQIVDDFREYVELCFWEF	174
CroshSGD	-----GGVNKDGVKFYHDFIDELLANGIKPFATLFHWDLPQALEDEYGGFLSDRIVEDFTEYAEFCFWEF	203
CroSGD	-----GGVNKDGVKFYHDFIDELLANGIKPFATLFHWDLPQALEDEYGGFLSDRIVEDFTEYAEFCFWEF	203
RseSGD	-----AGVNKDGVKFYHDFIDELLANGIKPSVTLFHWDLPQALEDEYGGFLSHRIVDDFCEYAEFCFWEF	195
RveSGD	-----AGVNKDGVKFYHDFIDELLANGIKPFATLFHWDLPQALEDEYGGFLSHRIVDDFCEYAEFCFWEF	195

Consensus	GDKVKxWTTFNEPHTFxxxGYAxGEFAPGRGxxx----------------------------xxGxPGTE	
UtoSGD	GDRVKYWMTFNETWSYSLFGYLLGTFAPGRGSTNEEQRKAIAEDLPSSLGKSRQAFAHSRTPRAGDPSTE	245
HpaSGD	GDRVKNWITLNEPWSFSTGGYVNGTFAPGRGASASEQV-NHDHIVPSRCS----AHVRQCISSNGNPGTE	207
MspSGD1	GDRVKHWITLNEPWSFSVGGYVNGTFAPGRGASSSDQENDHPAALPSRCS----PWQSQSISSNGNPGTE	240
CroshSGD	GDKVKFWTTFNEPHTYVASGYATGEFAPGRGGAD----------------------------GKGNPGKE	245
CroSGD	GDKVKFWTTFNEPHTYVASGYATGEFAPGRGGAD----------------------------GKGEPGKE	245
RseSGD	GDKIKYWTTFNEPHTFAVNGYALGEFAPGRGGKG----------------------------DEGDPAIE	237
RveSGD	GDKIKYWTTFNEPHTFTANGYALGEFAPGRGKNG-----------------------------KGDPATE	236

Consensus	PYxVTHNxLLAHKAAVExYRxxFQK---CQxGEIGIVLNSxWMEPLNEx-xADIDAxKRALDFMLGWFxE	
UtoSGD	PYIVTHNQLLAHAAAVKLYRFAYQNAQNAQKGKIGIGLVSIWAEPHNDT-TEDRDAAQRVLDFMLGWLFD	314
HpaSGD	PYLVTHNQLLAHAAAVKLYKSKFQK---PQEGKIGITLVSQWLEPLDENNDADVQASKRALDFMLGWLMQ	274
MspSGD1	PYVVTHNQLLAHAAAVELYKSNFQK---SQNGKIGITLVSQWMEPLDENSKADVEAAKRALDFMLGWFME	307
CroshSGD	PYIATHNLLLSHKAAVEVYRKNFQK---CQGGEIGIVLNSMWMEPLNET-KEDIDARERGLDFMLGWFIE	311
CroSGD	PYIATHNLLLSHKAAVEVYRKNFQK---CQGGEIGIVLNSMWMEPLNET-KEDIDARERGLDFMLGWFIE	311
RseSGD	PYVVTHNILLAHKAAVEEYRNKFQK---CQEGEIGIVLNSMWMEPLSDV-QADIDAQKRALDFMLGWFLE	303
RveSGD	PYLVTHNILLAHKAAVEAYRNKFQK---CQEGEIGIVLNSTWMEPLNDV-QADIDAHKRALDFMLGWFIE	302

```
Consensus  PLTTGXYPKSMRXLVGXRLPXFSXEXSEKLXGCYDFIGMNYYTXTYVXNAXKSX--XXXXYETDXXXX-X
UtoSGD     PVVFGRYPESMRRLLGNRLPEFKPHQLRDMIGSFDFIGMNYYTTNSVANLPYSR---SIIYN--------  373
HpaSGD     PL--------------------------------------------------------------------  276
MspSGD1    PLTTGDYPKTMRKLVGSRLPKFSAEQSKQLKGSYDFLGLNYYTADYVTSASSSTTGGNLSYTTDSQVT--  375
CroshSGD   PLTTGEYPKSMRALVGSRLPEFSTEDSEKLTGCYDFIGMNYYTTTYVSNADKIP--DTPGYETDARINKN  379
CroSGD     PLTTGEYPKSMRALVGSRLPEFSTEVSEKLTGCYDFIGMNYYTTTYVSNADKIP--DTPGYETDARINKN  379
RseSGD     PLTTGDYPKSMRELVKGRLPKFSADDSEKLKGCYDFIGMNYYTATYVTNAVKSNS-EKLSYETDDQVT-K  371
RveSGD     PLTTGDYPKSMREIVKGRLPRFSPEDSEKLKGCYDFVGMNYYTATYVTNAAKSNS-EKLSYETDDHVD-K  370

Consensus  XFXXXXDXXXVPIGXXXYGXWQHVVPXGLYKLLVYTKEXYHVPVIYVTEXGXVEENXTXI---------L
UtoSGD     ---PDSQAICYPMGEEAGSSWVYIYPEGLLKLLLYVKEKYNNPLIYITENGIDEVNDENL---------T  431
HpaSGD     ----------------------------------------------------------------------  276
MspSGD1    ---HTTDRNGVPIGPQGGSEWLHIYPEGIRKLLVYVKKTYNVPLIYITENGVDEVNDTSL---------T  433
CroshSGD   IFVKKVDGKEVRIGEPCYGGWQHVVPSGLYNLLVYTKEKYHVPVIYVSECGVVEENRTNILLTEGKTNIL  449
CroSGD     IFVKKVDGKEVRIGEPCYGGWQHVVPSGLYNLLVYTKEKYHVPVIYVSECGVVEENRTNILLTEGKTNIL  449
RseSGD     TFER----NQKPIGHALYGGWQHVVPWGLYKLLVYTKETYHVPVLYVTESGMVEENKTKI---------L  428
RveSGD     TFDRVVDGKSVPIGAVLYGEWQHVVPWGLYKLLVYTKETYHVPVLYVTESGMVEENKTKI---------L  431

Consensus  LXEARXDXXRXDYXQXHLASVRDAIXDGVNVKGYFVWSFXDNFEWXXGXXXRXGXIHVDYX-XFXRYPKX
UtoSGD     MWEALYDTQRISYHKQHLEATKQAISQGVDVRGYYAWSFTDNLEWASGFDSRFGLNYVHFGRKLERYPKL  501
HpaSGD     ----------------------------------------------------------------------  276
MspSGD1    LSEARVDNTRIKYIQDHLLNIRLAISDGVNVKGYFVWSLLDNFEWSEGYTVRFGFIHIDYTNNFARYPKD  503
CroshSGD   LTEARHDKLRVDFLQSHLASVRDAIE--------------------------------------------  475
CroSGD     LTEARHDKLRVDFLQSHLASVRDAIDDGVNVKGFFVWSFFDNFEWNLGYICRYGIIHVDYK-TFQRYPKD  518
RseSGD     LSEARRDAERTDYHQKHLASVRDAIDDGVNVKGYFVWSFFDNFEWNLGYICRYGIIHVDYK-SFERYPKE  497
RveSGD     LSEARRDPERTDYHQKHLASVRDAIDDGVNVKGYFVWSFFDNFEWNLGFIGRYGIIHVDYN-SFERCPKE  500

Consensus  SAIWXKNFXXXXXXXXX-XXXXXXXX---XLVKXXXX------------------
UtoSGD     SAGWFKFFLENGKSASFCWSIIGNNI---CLNKRSRCTLVDCRIYILLVIRIYVC  553
HpaSGD     -------------------------------------------------------  276
MspSGD1    SAIWFMNSFHKEYPKKFLKRTLEDHED---FVSKKRLRQ----------------  539
CroshSGD   -------------------------------------------------------  475
CroSGD     SAIWYKNFISEGFVTNTAKKRFREEDKLVELVKKQKY------------------  555
RseSGD     SAIWYKNFIAGKSTTSP-AKRRREEA-QVELVKRQKT------------------  532
RveSGD     SAIWYKNFIAGVSTTSP-AKRRREEAEGVELVKRQKT------------------  536
```

Tetrahydroalstonine synthase (THAS)

```
Consensus           -------------------------------XXXXSXEXXXXVKXXGXAAXDXSGXXSPFKFXRRATXEX

RteTHAS5            ------------------------------MVKAIRVHELGGPEVLKWEDVE-----------IGEPKEG  29
CroTHAS2            ----------------------------------MSSKSAKPVEAYGWAAKDTSGLLSPFKFLRRTTGEH  36
CroTHAS3            -------------------------------MAVPSAETGKTIEAYGWAARDSSGLLSPFKFQRRATTEH  39
RteTHAS2            ---------------------------------MAAAETAKTIEAYGWAARDTSGVLSPFKFQRRATAEH  37
RteTHAS             ---------------------------------MAAAETAKKIEAYGWAARDTSGVLSPFKFQRRATAEH  37
RteTHAS3            ---------------------------------MAAAETAKKIEAYGWAARDTSGVLSPFKFQRRATAEH  37
RteTHAS4A           MCSSGRTIKESSVPSRIGGQINSAELIACFIMAPNSPEDAHPVKTFGLAARDSSGLFSPFKFSSRATGER  70
RteTHAS4B           -------------------------------MAPNSPEDVHPVKTFGLAAKDSSGLFSPFKFSRRATGER  39
CroTHAS1            ----------------------------MAMASKSPSEEVYPVKAFGLAAKDSSGLFSPFNFSRRATGEH  42
RteYOS              ------------------------------MAS-ESPEDVYPVKTIGLAAKDSSGVFSPFKFSRRATGEN  39
RteAMS              ---------------------------MADEMSAKSPEDAHSTKTFGLAAMDSSGVFSLFKFSRRATGEH  43
CroHYS              -------------------------------MAAKSPENVYPVKTFGFAAKDSSGFFSPFNFSRRATGEN  39
CroTHAS4            -------------------------------MAAKSPENVYSVKTFGFAAKDSSGLFSPFNFSRRATGEN  39
UtoHYS              ----------------------------------------------------------------------
UtoTHAS             ----------------------------------------------------------------------
MspTHAS             --------------MAGENRSNVFGLATRKISLEIEISGENRVKTFGWAAKDTSGVLCPFDFTRRATGDE  56
MSTRG5530(Rte-YOS)  -------------------------------MARKSPEEEHPVKAYGWAARDSSGVLSPFKFSRRATLED  39
MSTRG5531(Rte-YOS)  -------------------------------MAAKSPEEEHPVKAYGWAARDSSGVLSPFKFSRRATLED  39
HpaTHAS2            ----------------------------------------------------------------------
HpaTHAS             ----------------------------------------------------------------------
MSTRG5534(Rte-YOS)  --------------------------------MAKSPEVEHPVKAFGWAARDPSGVLSPFNFSRRATGEH  38


Consensus           DVQXKVLYCGXCXXDXXXXKNXFGXTXYPXVXGHEXVGVVTEVGXKVXKFKVGDKVGVGXXVGXCRXCXX

RteTHAS5            EIKVINKAIGLNFIDTYFRKGVYKAATMPFTPGMEAVGVVTAVGPGLTGRKVGDVVAYAGN---------  90
CroTHAS2            DVQFKVLYCGLCDWDVITTKNTYGTTKYPFVPGHEIMGIVTEIGNKVKKFKVGDKVGVGNFIGSCGKCER  106
CroTHAS3            DVQLKILYCGMCDWDLHVVKNWFGTTNYPIVPGHEAVGVVTEIGNKVQKFKIGDIVGVSTYIRTCRSCER  109
RteTHAS2            DVQLKVLYCGMCDWDSIVVKNGFGTTKYPIVPGHEVVGVVTEIGSKVQKFKVGDIVGVSSYVRTCRKCKR  107
RteTHAS             DVQLKVLYCGMCDWDSVVVKNGFGTTKYPIVPGHEVVGVVTEIGSKVQNFKVGDVVGVSSYVRTCRKCKR  107
RteTHAS3            DVQLKVLYCGLCDWDSVVVKNGFGTTKYPIVPGHEVVGVVTEIGSKVQKFKVGDIVGVSSYVRTCRKCKR  107
RteTHAS4A           DVQFKVLYCGTCNYDAAASKNKFGTTTYPFVLGHEAVGVVTEVGAKVRKFKVGEKVGVGGVVGACRICEM  140
RteTHAS4B           DVQFKVLYCGICNYDAAASKNKFGMTTYPFVLGHEAVGVVTEVGAKVRKFKVGEKVGVGGVVGACRICEM  109
CroTHAS1            DVQLKVLYCGTCQYDREMSKNKFGFTSYPYVLGHEIVGEVTEVGSKVQKFKVGDKVGVASIIETCGKCEM  112
RteYOS              DVQFKVLYCGICQYDREMSRNKFGMTNYPYVFGHEIVGVVTEVGCKVQKFVVGDKVGIGSITDTCGECEM  109
RteAMS              DVQLKVLYCGTCKYDWEMSRNKFGMTKYPFVIGHEIVGVVTEVGWKVQKFKAGDKVGVGGFVGTCGKCEM  113
CroHYS              DVQFKVLYCGTCNYDLEMSTNKFGMTKYPFVIGHEIVGVVTEIGSKVQKFKVGDKVGVGGFVGACEKCEM  109
CroTHAS4            DVQFKVLYCGTCYYDWAMITNKYGMTNYPFVIGHEIIGVVTEVGSKVKKFKVGDKVGVGGHVGACEKCEL  109
UtoHYS              ----------------------------------------------------------------------
UtoTHAS             ------------------------------------------------KCKVGDVVGVGALIGSCRTCHS  22
MspTHAS             DVRFKILYCGICHSDLHQAKDDFGFGIYPLVPGHEIAGIVTEVGSKVTKFKAGDKVGVGTIVGSCRSCNR  126
MSTRG5530(Rte-YOS)  DVRLKVHYCGVCHTDLHFLKNDWGFSTYPLVPGHEIVGEVTEVGKKVTKVKVGDKVGVGCLVGSCRACDR  109
MSTRG5531(Rte-YOS)  DVRLKVLYCGVCHTDIHFLKNEWGFSTYPFVPGHEIIGEVIEVGSKVTKVKVGDKVAHGGIIGSCRTCDN  109
HpaTHAS2            -------------------------------------------------GKVGDVVGVGCLVGSCRNCDM  21
HpaTHAS             ------------------------------------------------KVKVGDEVGVGCLVGSCRNCNR  22
MSTRG5534(Rte-YOS)  DVQFKVLYCGICHSDLHMIKNEWGITKYPIVPGHEIVGVVTEVGSKVENFKVGDKVGVGCLVGSCRKCDM  108
```

```
Consensus             CXXXXEXYCPXXXXTDGXXXXDG----------------XXXYGGXSNIMVXXEXFVVRWPENLPLDXGV

RteTHAS5              -----------------------------------------PMGAYAEEQILPADRAVPVPPSIDPIVGA   119
CroTHAS2              CNEGLEPYCPKVIYTDGTAFSDENNTVYGDVSGD---GEDRIYGGYSNIMVANEYVVFRWPENLPLAAGV   173
CroTHAS3              CKEGEDSYCPSLITGDGTSFSDGNDVFFYDPNDDNTKETTKTYGSYSNFTVVDEYYVIRWPENFPLAAGV   179
RteTHAS2              CKEGLDSYCPNLITGDGTSFSDGNDLYFHDPNDTE----SKIYGGFSNITVVEEYYVVRWPENFPLAAGV   173
RteTHAS               CKEGFDSYCPNLITADGTSFSDGNDIYFHDPNDTE----SKIYGGFSNITVVDEYYVVRWPENFPLAAGV   173
RteTHAS3              CKEGFDSYCPNLITADGTSFSDGNDLYFHDPNDTE----SKIYGGFSNITVVDEYYVVRWPENFPLAAGV   173
RteTHAS4A             CADEVEQYCPKAVATDG---FMG----------------SGQYGACSDIMVADEDFLVLWPENLPLDSGV   191
RteTHAS4B             CANEVEQYCPKAVATDG---FMG----------------SGQYGSCSDIMVADEDFLVLWPENLPLDCGV   160
CroTHAS1              CTNEVENYCPEAGSIDS------------------------NYGACSNIAVINENFVIRWPENLPLDSGV   158
RteYOS                CTNEVENYCPKAVSIDS---NFA----------------N-NYGGCSNIMVTKENFVIHWPENLPLDSGV   159
RteAMS                CIIQAENNCPKLESTDG---HFG----------------T-NYGGCSDIMVADEKFVVLWPENLPPDSGV   163
CroHYS                CVNGVENNCSKVESTDG---HFG----------------N-NFGGCCNIMVVNEKYAVVWPENLPLHSGV   159
CroTHAS4              CINGVENNCPEAESTDG---FSG----------------K-NFGGCSNIMVVNEKYAVVWPENLPLHSGV   159
UtoHYS                ----------------------------------------------------------------------
UtoTHAS               CTNDLENYCPKAVLTE------------------------ATSGGCSNFVVADEDFVFRWPEKLPLDLGA   68
MspTHAS               CSNDLEPYCPKMMMTYNSIDEDG----------------IPTRGGFSNEMVVNEHFAVRWPDNLPLDGGA   180
MSTRG5530(Rte-YOS)    CSADLENYCPKQVPTYSMPYFDG----------------TITYGGYSNEMVCNEHFIIRFPNKIPLDAGA   163
MSTRG5531(Rte-YOS)    CRADMESYCPKMVMAHGSPNFDG----------------TITYGGFSNEMVLNEHFVIRYPDNLPLAAGA   163
HpaTHAS2              CSKDLENYCPGQILTYSAAYTDG----------------TITYGGYSDLMVADEHFVIRWPENFPLDAGR   75
HpaTHAS               CNEDLENYCPAQIFTYGSPDTDG----------------TPTYGGYSDIMVVDEHFVVRWPENFPLDAGA   76
MSTRG5534(Rte-YOS)    CSNDLENYCPGQILTYSATNTDG----------------TTTYGGYSDLMVADEHFVIRWPENLPMDIGA   162


Consensus             PLLCAGITXYSPM-RXFGLDKPGXHXGVXGLGGLGHXAVKFAKAFGAKVTVISSSXXKKXEAIEKXGADX

RteTHAS5              SILLKGMTAQFLVRHCFKVECGHTVLVHAAAGGVGSLLCQWANALGATVIGTVSTKEKAAQ-AKDDGCHH   188
CroTHAS2              PILCGGIVPYSPM-RHFGLDKPGLSIGVVGFGRIGKLAVKFAKAFGANVTVISTSISKKQEAIEKYGVDR   242
CroTHAS3              PLLCAGTVPYSPM-RHFGFDKPGIHIGVVGFGGIGKLVVKFAKAFGVKVTVISTSIDKKHEAIHEYGAHG   248
RteTHAS2              PVLCAGTVPYSPM-RCFGFDKPEIHLGVVGLGGIGRLTVKFAKAFGAKVTVISTSIDKKQEAIEKYGADS   242
RteTHAS               PLLCAGTVPYSPM-RCFGFDKPEIHLGVVGLGGIGKLTVKFAKAFGAKVTVISTSIDKKQEAIEKYGADR   242
RteTHAS3              PLLCAGTVPYSPM-RCFGFDKAEIHLGVVGLGGIGKLTVKFAKAFGAKVTVISTSIDKKQEAFEKYGADR   242
RteTHAS4A             PLLC-GVTIYSPM-RRFGLDKPGTHIGIAGLGGFGHMAVRFAKAFGTQVTVISSSAKKKRETLEKFGADS   259
RteTHAS4B             PLLC-GVTIYSPM-RRFGLDKPGTHIGIAGLGGFGHMAVRFANAFGTKVTVISSSAKKKRETLEKLGADS   228
CroTHAS1              PLLCAGITAYSPM-KRYGLDKPGKRIGIAGLGGLGHVALRFAKAFGAKVTVISSSLKKKREAFEKFGADS   227
RteYOS                PLLCAGINAYSPM-RRYGLDKTGMRVGIAGLGGLGHVGVRFAKAFGAKVTVISSSLKKKHEALEKFGADS   228
RteAMS                PLLCAGITTYSPM-RRYGLDKPGMRVGIAGLGGLGHLAVRFAKAFGANVTVISSSLEKKREALEKFGADS   232
CroHYS                PLLCAGITTYSPL-RRYGLDKPGLNIGIAGLGGLGHLAIRFAKAFGAKVTLISSSVKKKREALEKFGVDS   228
CroTHAS4              PLLCAGITTYSPL-RRYGLDKPGLNIGIAGLGGLGHLAIRFAKAFGAKVTLISSSVKKKREALEKFGVDS   228
UtoHYS                -------------------------------------SIRVAKAFGAKVTVISSTPGKKEEAIKNFKADS   33
UtoTHAS               PLLCAGITTYSPL-KNFGLDKPGLHVGVAGLGGLGHVAVKFLKAFGAKVTVISSSDNKKKEAIEKYGADA   137
MspTHAS               PLLCAGSTVYSPM-MYYGLSKPGTHLGVAGLGGLGHVAVKFAKAFGVEVTVISTSPSKKEEAINHLGADH   249
MSTRG5530(Rte-YOS)    PLLCAGITVYSTM-KHYGIAKAGNHIGVNGLGGLGHVAVKFAKAMGAKVTVISTSGSKKDEAINLLGADA   232
MSTRG5531(Rte-YOS)    PLLCAGITVYSPM-KYYGIAKPGNHIGVNGLGGLGHMAVKFAKALGAKVTVISSSESKKDDSINRLGADA   232
HpaTHAS2              PLLCAGITTYSPL-RYFGLDKPGTHVGVVGLGGLGHVAVKFAKAFGAKVTVISTSEGKKKEAIEKLGADA   144
HpaTHAS               PLLCAGITTYSPL-RYFGLDKPGIHIGVVGLGGLGHVAVKFAKAFGAKVTLISTSASKKQDAIEKLGADS   145
MSTRG5534(Rte-YOS)    PLLCAGITTYSPL-RYFGLDKPGTHVGIVGLGGLGHVAVKFAKAFGAEVTVISTSESKKQEAVEKLGADA   231
```

```
Consensus              FLxSxxPExMQAAxGT-----LDGIIDTVPxxHPIxPLLxLLKxxGKLIxLGAPx-xPxELPxxxLLMGR

RteTHAS5               VIIYKDEDFVTRVNEITSGKGVEVVYDSV-GKDTFEGSLACLKYRGYMVNFGQSSGMPDPVPLSAIAAKS   257
CroTHAS2               FLISKEPEEMKAAEST-----LDGIFDCVPSVHPLHPLLNLLKFEGTFVMLGVAV-EAYELPVSPLLMGR   306
CroTHAS3               FLLSKEPQQLQAAINT-----MEGIVDTVPKVHPILPLIKLLKFDGTLLMLGAPP-EPYEFPISTLLMGR   312
RteTHAS2               FLLSKEPEQLQAAVDT-----LDGIIDTVPRIHPILPLIKLLKFDGTLVLLGAPL-EPYELPVSPLLMGR   306
RteTHAS                FLLSKEPEQLQAAADT-----LDGIIDTVPRTHPILPLIKLLKFDGTLVLLGAPL-EPYELPVSPLLMGR   306
RteTHAS3               FLLSKEPEQLQAAADT-----LDGIIDTVPRIHPILPLIKLLKFDGTLVLLGAPL-VPYELPVSPLLMGR   306
RteTHAS4A              FLVSTNPEKMQAAAGT-----LDAIIDTIPVVHPLEPLLALLKPFGKLITICAVD-APCQLYTGALLQGG   323
RteTHAS4B              FLVSTNPEKMQAAAGT-----LDAIIDTIPVVHPLEPLLALLKPFGKLITICAVD-APCQLYTGALLQGG   292
CroTHAS1               FLVSSNPEEMQGAAGT-----LDGIIDTIPGNHSLEPLLALLKPLGKLIILGAPE-MPFEVPAPSLLMGG   291
RteYOS                 FLVSSDPEEMEAAAGT-----LDGIIDTVPADHSIEPLLALLKPLGKLIILGAPE-RPFEVPAPALLQGG   292
RteAMS                 FLVSSDLEQLQSAAGT-----LDGIIDTMPVFHPIEPFLALLKPLGKLIILGVPE-KPFEVPAPALLMGG   296
CroHYS                 FLLNSNPEEMQGAYGT-----LDGIIDTMPVAHSIVPFLALLKPLGKLIILGVPE-EPFEVPAPALLMGG   292
CroTHAS4               FLLNSNPEEMQGAYGT-----LDGIIDTMPVAHSIVPFLALLKPLGKLIILGVPE-EPFEVPAPALLMGG   292
UtoHYS                 FLVSRDTEAMQAAAGT-----LHGILDTVSAVHPILPLLSLLKNHSKYIMLGAPA-KPLELPVFPMLMGR   97
UtoTHAS                FLVSSNPEQMQAAAGT-----LSGILDTIPSAHSLLPLLNLLLPHGKLIVLGAPI-EPFVLPIYPLLQGG   201
MspTHAS                FLVSTDQDQMQAAMGT-----LDGIIDTISAVHPIMPLFGLLKHDAKLIVVGVQN-KPLEVDIPALIIGR   313
MSTRG5530(Rte-YOS)     FLLSKNQEELQAATGT-----LDGIIDCVSAKHPIMPLLDLLKFEGKLCMVGAPA-EPLELQSAPLLVGR   296
MSTRG5531(Rte-YOS)     FLLSKNPEELQAATGT-----LDGIVDCVSAKHPIIPLLGLLKSQGKLVMVGAPG-EPLELHSAPLLMGR   296
HpaTHAS2               FLNSRDPEQMQAARGT-----IDGIIDTVSATHPVIPLINLVKSHGKIILXGAGE-KPLEVPAFPLLIGR   208
HpaTHAS                FILSNDPKEMEAAFGT-----MDGIIDTVSAVHSILPLIDLLAFDGKLVEVGIPA-KPLELPIYNLSIGR   209
MSTRG5534(Rte-YOS)     FLVSRDPDQMQAAAGS-----MDGIIDTVSAVHPVMPLVNLLKSHGKLILVGAPE-KPLELPVFPLLVGR   295
```

```
Consensus              KxxxGSxxGSMKETQEMxDFAAKHNIVADVExIPxDYxNTA----xE---RxxKxDVKYRFVIDVG----

RteTHAS5               LFLT---------RPSMMHYTLSREELL--EAAGEVFANVASGV------LRVRVNHTYRLSEAVQAHSD   310
CroTHAS2               RKFVGSISGTMKETQEMLDFAAKHNIVSDIELIPMDYVNTA----LE---RIAKGNHKDAFVIDIE----   365
CroTHAS3               KRVVGSAGASMKETQEMMDFAAKHNIVADVEIIPIDYANTA----IE---RIEKGDFKNRFVIDIE----   371
RteTHAS2               KRVVGSAGASMKETQEMMDFAAKHNIVADVEIIPMNYANTA----IE---RIEKGDFKNRFVIDIE----   365
RteTHAS                KRVVGSAGASMKETQEMMDFAAKHNIVADVEIIPMDYASTA----IE---RIEKGDFKNRFVVDIG----   365
RteTHAS3               KRVVGSAGASMKETQEMMDFAAQHNIVADVEIIPVDYASTA----IE---RIERGDFKNRFVVDIG----   365
RteTHAS4A              KSIVGSSHASMKDTQEMIDFAAKHNIVADVEVIPIDYVNAA----MK---RLEKSDVKYRFVIDVG----   382
RteTHAS4B              KSIVGSSHASMKDTQEMIDFVAKHNIVADVEVIPIDYVNAA----MK---RLEKSDVKYRFVIDVG----   351
CroTHAS1               KVMAASTAGSMKEIQEMIEFAAEHNIVADVEVISIDYVNTA----ME---RLDNSDVRYRFVIDIG----   350
RteYOS                 KLMAASTSGSVKELQEMVDFAAKHNIVADVEIIPIDYVNTA----MD---RLDKSDVKYRFVIDMG----   351
RteAMS                 KLMAGSSSGSMKETQELLDFAAKHNITADVDVIPIDYVNTA----MK---RIAKSDVKYRFVIDVG----   355
CroHYS                 KLIAGSAAGSMKETQEMIDFAAKHNIVADVEVIPIDYLNTA----ME---RIKNSDVKYRFVIDVG----   351
CroTHAS4               KLIAGSAAGSMKETQEMIDFAAKHNIVADVEVIPIDYLNTA----IE---RIKNSDVKYRFVIDVG----   351
UtoHYS                 KIVAGSNIGGIKETEEMLKFAA------------------------------------------------   119
UtoTHAS                RVVAGSATGGLKQIQEMLDFSA------------------------------------------------   223
MspTHAS                KMLGASAVAGMKETQEMIDFAAKHNITADIEVISMDYVNKA----ME---RLEKGDVRYRFVIDVG----   372
MSTRG5530(Rte-YOS)     KTIAGNSIGGLKETQEMIDFAAKHNITADIELISIDNINKA----FE---RLAKGDVRYRIVIDVA----   355
MSTRG5531(Rte-YOS)     KMIGGSDAGGMKEIQEMVDLAAKHNITADVELVSMDDINTV----VE---RLVRGDVRYRFVIDVA----   355
HpaTHAS2               KTLTGSAIGRSEGNSGDAGLRSKAQYLGRC*GHPNGLCQHCNGAPLES*CQIPLCDRHWEFIE-VS----   273
HpaTHAS                KIVGATTIGGLKETQEMIDFAVKHNIVPDVEIIPIDYVNTA----ME---RLERGEVKYRFVIDVG----   268
MSTRG5534(Rte-YOS)     KIIGGSAIGGLKETQEMIDFAAKNNILPDVELIPMDYVNTA----ME---RLLKADVKYRFVIDIG----   354
```

**Consensus**	**- - N T L K S** X X - - - - - - - - - - -	
RteTHAS5	L E N R R T A G S V V L I P D T I D Q *	330
CroTHAS2	- - N T L K S A - - - - - - - - - - - -	371
CroTHAS3	- - N S L G S V - - - - - - - - - - - -	377
RteTHAS2	- - N T L K S A - - - - - - - - - - - -	371
RteTHAS	- - N T L K S A - - - - - - - - - - - -	371
RteTHAS3	- - N T L K S A * - - - - - - - - - - -	372
RteTHAS4A	- - N T L K S A * - - - - - - - - - - -	389
RteTHAS4B	- - N T L K S A * - - - - - - - - - - -	358
CroTHAS1	- - N T L K S N - - - - - - - - - - - -	356
RteYOS	- - N T L R S A * - - - - - - - - - - -	358
RteAMS	- - N C L K S A * - - - - - - - - - - -	362
CroHYS	- - N T L K S P S F - - - - - - - - - -	359
CroTHAS4	- - N T L K S P S F - - - - - - - - - -	359
UtoHYS	- - - - - - - - - - - - - - - - - - - -	119
UtoTHAS	- - - - - - - - - - - - - - - - - - - -	223
MspTHAS	- - N T L V A T T S T - - - - - - - - -	381
MSTRG5530(Rte-YOS)	- - N T L R A P * - - - - - - - - - - -	362
MSTRG5531(Rte-YOS)	- - N T L K A P * - - - - - - - - - - -	362
HpaTHAS2	- - - - L - - - - - - - - - - - - - - -	274
HpaTHAS	- - N T L K S T * - - - - - - - - - - -	275
MSTRG5534(Rte-YOS)	- - N T L K S A * - - - - - - - - - - -	361

Supplementary material 4.

SA concentration experiment

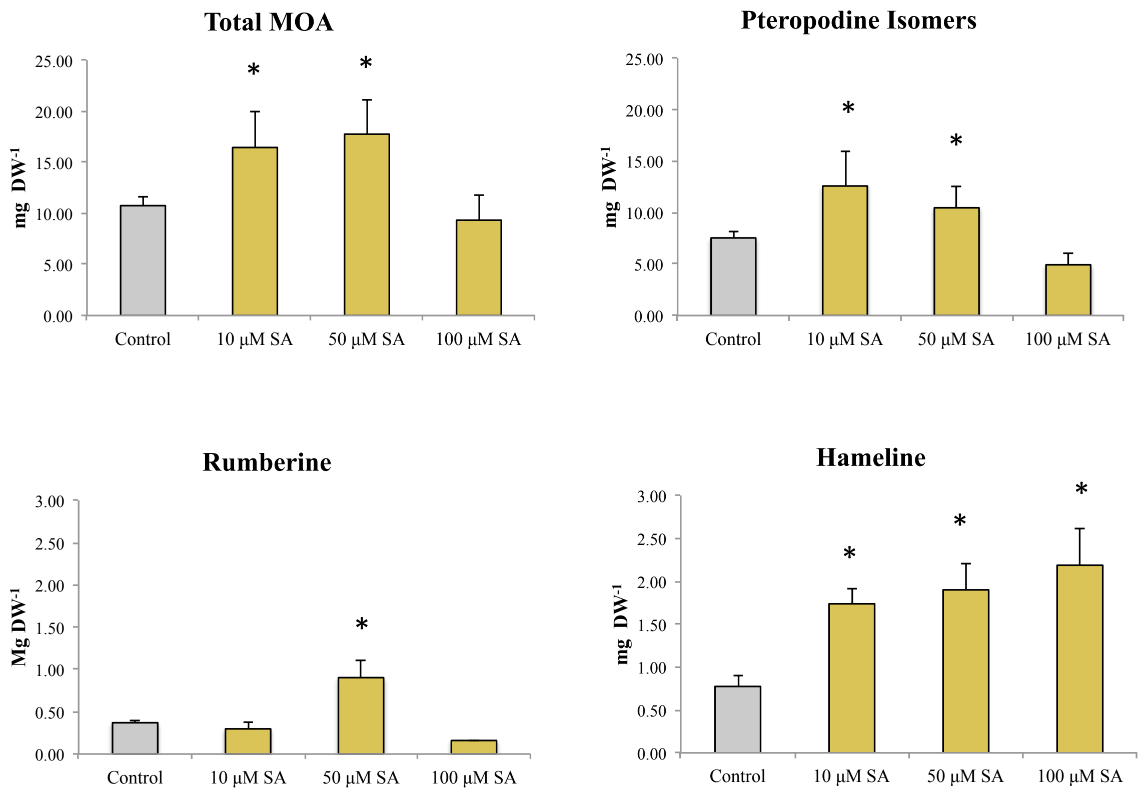

Figure S2. MOA production in 1.5-month-old *H. patens* plants, under different SA treatments after 120 h (n=3).

Supplementary material 5.

HPLC chromatogram of the *H. patens* extract indicating the retention times of each MOA.

Alkaloid detection was at 244 nm. Retention times: [1] rumberine 9.4 min, [2] speciophylline 13.5 min, [3] uncarine F 16.5 min, [4] hameline 20 min, [5] pteropodine 23.2 min, [6] isopteropodine 40.9 min.

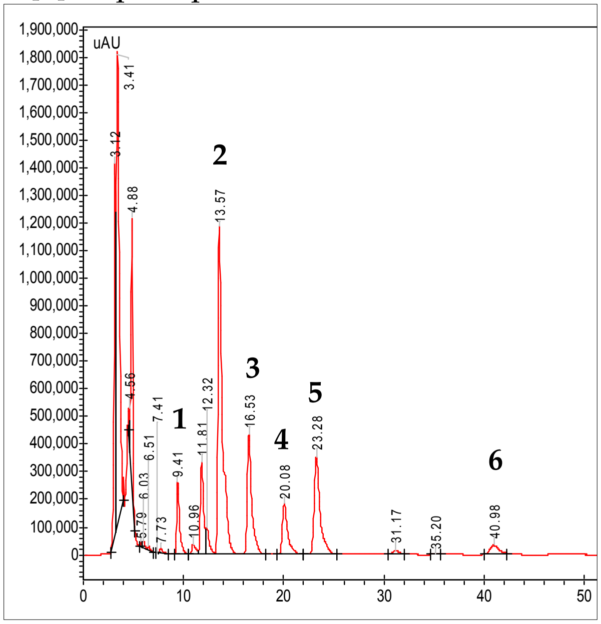

Supplement: Supplementary file 1 [file plants-13-00966-s001.zip › plants-2868229-supplementary.pdf]
